# Technical guidelines for risk assessment of heavy metals in traditional Chinese medicines

**DOI:** 10.1186/s13020-023-00771-3

**Published:** 2023-06-07

**Authors:** Tian-Tian Zuo, Lei Zhang, Ying Wang, Li-Xing Nie, Ming-Rui Shen, Li-Na Liu, Jian-Dong Yu, Hong-Yu Jin, Feng Wei, Shuang-Cheng Ma

**Affiliations:** 1 National Institutes for Food and Drug Control, No. 31 Huatuo Road, Daxing District, Beijing, 102629 China; 2 WHO Collaborating Center for Herbal Medicine (CHN-139), Beijing, China; 3 China National Center for Food Safety Risk Assessment, Beijing, 100082 China; 4 Chinese Pharmacopeia Commission, Beijing, 100061 China

**Keywords:** Traditional Chinese medicine, Guideline, Risk assessment, Heavy metals

## Abstract

**Background:**

Heavy metals are considered a global concern because they can deteriorate human health. This guideline aims to scientifically evaluate health risk of heavy metals in TCM and to propose a reference for decision making in developing TCM-related health policies.

**Methods:**

Using a multidisciplinary approach, a steering committee oversaw the development of the guideline. To obtain a reasonable and accurate risk assessment, key exposure assessment parameters for TCM, e.g., exposure frequency (EF), exposure duration (ED), and daily ingestion rate (IR) were obtained from surveys. In addition, transfer rates for heavy metals from Chinese medicinal materials (CMM) to decoctions or preparations were examined.

**Results:**

Based on the scientific theory of risk control, the guideline was designed systematically, and principles and procedures for the risk assessment of heavy metals in TCM were identified. The guideline can be utilized to assess the risk of heavy metals in CMM and Chinese patent medicines (CPM).

**Conclusion:**

This guideline may help standardize the risk assessment of heavy metals in TCM, advance regulatory standards for heavy metals in TCM, and ultimately improve human health through scientific TCM usage in clinic.

**Supplementary Information:**

The online version contains supplementary material available at 10.1186/s13020-023-00771-3.

## Background

With the ever-increasing popularity of Traditional Chinese medicine (TCM) and the global expansion of the TCM market, safety has become a major concern for both public health institutions and the general population [1]. TCM products include Chinese medicinal materials (CMM) and Chinese patent medicines (CPM). CMM are natural products found in the environment, with potential contamination by heavy metals during the processes of cultivation, harvesting, and processing. CPM are preparations composed of different types of CMM, thus heavy metals in CMM are likely to be transferred to CPM, resulting in their contamination. Heavy metal contamination represents a serious threat, as they are ubiquitous in the environment, non-biodegradable, persistent and toxic, even at extremely low levels [2–13]. The heavy metal problem has become a major factor affecting the quality and safety of TCM, attracting the attention of both society and regulatory agencies [14–17].

International organizations such as the Food and Agriculture Organization of the United Nations/World Health Organization (FAO/WHO), European Food Safety Authority (ESFA), Agency for Toxic Substances and Disease Registry (ATSDR), United States Environmental Protection Agency (US EPA), and Agency for Toxic Substances and Diseases Registry (ATSDR) have assessed the safety of food-related chemicals for a long time [18–20]. Risk assessment represents the central scientific part of risk analysis, and primarily addresses the need for optimal decisions for preventing disease and improving public health. Risk assessment of chemicals can be generally described as characterizing the potential hazards and the associated risks to life and health after human exposure for a given time period. However, TCM are different from food: they are used for the treatment of diseases and are less frequently consumed compared with food. Therefore, to develop a risk assessment model suitable to TCM, conventional models should be refined and a guideline readily applicable to the characteristics of TCM developed by regulatory authority is urgently needed.

## Methods

Guideline design and development were performed in accordance with the “World Health Organization Handbook for Guideline Development” , under the direction of Dr. Shuangcheng Ma, the Chairman of WHO Collaborating Center for Herbal Medicine. The research was approved by National Medical Products Administration (NMPA).

### Before drafting

Several aspects were considered before drafting the guideline. Firstly, risk assessment of heavy metals in TCM should be based on the fact that TCM are natural products. During the modernization of TCM, centralized and large-scale artificial cultivation is inevitable. Thus, it is impossible to achieve zero pollution or no residue. The main goals of risk assessment are to scientifically assess the health risk of heavy metals in TCM, to ensure the safe usage of TCM, and to guide the healthy development of the TCM industry. Secondly, TCM differs from food in terms of intake (exposure). Thus, it is suggested to establish exposure parameters that could reflect the characteristics of TCM, including exposure frequency (EF), exposure duration (Ed), daily ingestion rate (IR), in order to accurately evaluate the risk conferred by heavy metals in TCM. Thirdly, during risk assessment, one should consider the risk–benefit balance to determine acceptable risk levels for the different clinical purposes of TCM.

### Constitution of a steering committee and a multidisciplinary panel

The steering committee is the Professional Committee of Chinese Pharmacopoeia Commission for Safety and Quality Control of traditional Chinese medicine (TCM), consisting of 11 specialists and TCM experts in TCM, agriculture and food safety risk assessment. The roles of this committee were to design the research, to provide guidance and to perform a review of study contents and methodologies. The multidisciplinary panel comprised experts from drug control institutes, scientific researchers and public health policy makers, with the goal of establishing a representative and reliable survey and database of heavy metal contents in TCM. The panel was responsible for conducting the study.

### Systematic review and evaluation

Studies on health risk assessment of heavy metals were systematically reviewed. The principles and procedures established by various organizations, including FAO/WHO, ESFA, US EPA, and ATSDR, were reviewed and adopted as a foundation for the development of our risk assessment guideline. In addition, many databases, including CDSR, CMR, HTA, NHSEED, EMBASE, Ovid MEDLINE(R), China Journals Full-text Database and CNKI, were searched for relevant publications either in Chinese or English.

### Survey and questionnaires

For ensuring an accurate assessment of health risks related to TCM intake, a questionnaire-based TCM use survey was carried out in 11 Chinese provinces/cities, including Beijing, Liaoning, Heilongjiang, Jiangsu, Zhejiang, Shandong, Hubei, Guangdong, Chongqing, Yunnan, and Gansu. Totally 20,917 randomly selected volunteers (9420 males and 11,497 females) were enrolled. In terms of age, 72.63%, 19.51% and 7.86% of these individuals were 18–44, 45–59 and > 60 years old, respectively. Participant age averaged 37.8 years. The participants included 11,358 urban and 9559 rural residents. Both face-to-face and hospital prescription surveys were utilized for the identification of significant factors affecting heavy metal exposure.

The face-to-face survey was carried out by the multi-stage stratified cluster random-sampling method. The basic demographic information of the volunteers, ingestion purpose, ingestion mode, EF, ED, and IR were assessed. The hospital prescription survey was performed in four Beijing hospitals to assess TCM prescriptions. Totally 300 to 800 prescriptions were collected from each hospital. Patient gender, patient age, dosage form, TCM ingredients in each prescription, and administration mode were examined. After the survey, the data were collected by Epidata’s double entry to generate a database for further analysis and building risk assessment models for heavy metals in TCM.

#### Consumption of TCM by residents from different provinces

In this survey, 21.84% of respondents used TCM in the past 6 months, with higher reported usage in women (24.21%) compared with that in men (18.96%). There was a statistically significant difference (P < 0.01) in the rate of TCM usage among survey respondents from different provinces, with the highest reported rates in Guangdong Province (45.20%) and Jiangsu Province (31.18%), followed by Gansu Province (22.03%), Zhejiang Province (21.24%), and Shandong Province (20.75%). In contrast, the rate of TCM usage was between 10 and 20% in Heilongjiang Province, Liaoning Province, Yunnan Province, and Hubei Province, while Chongqing City (11.61%) and Beijing City (11.45%) had the lowest TCM consumption rates.

#### Consumption of TCM by different population categories

Consumption of TCM by residents of different ages, academic qualifications, occupations, educational levels, and incomes was shown in Table 1. From the survey, it could be concluded that there were several key factors affecting the consumption rate of TCM, involving gender, age, province of residence, and occupation. The results showed that women, older adults, retirees, and respondents from Guangdong Province were more likely to consume TCM. Additionally, the most common TCM consumption mode was decoction (49.10%). Less common modes of TCM consumption included soaking TCM in wine (2.49%) or taking TCM with food (1.91%).

**Table 1:** TCM used in different population categories

	Grouping variable	Total number of people	Number of users (%)
Sex	Male	9420	1786 (18.96%)
	Female	11,497	2783 (24.21%)
Place of residence	Urban	11,376	2457 (21.60%)
	Rural	9541	2112 (22.14%)
Age	18–44 years	15,193	2987 (19.66%)
	45–59 years	4080	1046 (25.64%)
	60 years and above	1644	536 (32.60%)
Academic qualifications	Primary school and below	3029	703 (23.21%)
	Junior high school	6583	1449 (22.01%)
	High school and technical secondary school	4582	970 (21.17%)
	College	2990	618 (20.67%)
	University and above	3733	829 (22.21%)
Occupation	Housekeeping	5185	1140 (21.99)
	Current student	137	20 (14.60%)
	Civil servants, enterprises and institutions	5353	1132 (21.15%)
	Service industry	6987	1566 (22.41%)
	Unemployed and others	2056	325 (15.81%)
	Retired	1177	378 (32.12%)
Annual income	Less than 10,000	4951	981 (19.81%)
	10,000–20,000	5978	1402 (23.45%)
	20,000–30,000	3291	712 (21.63%)
	30,000–40,000	2471	542 (21.93%)
	40,000 or more	2502	521 (20.82%)

#### Exposure frequency and daily ingestion rate

The results of the survey, including questionnaire on TCM exposure frequency and daily ingestion rate, were shown in Table 2 and Table 3. In addition, a total of 2193 TCM prescriptions were collected from 4 hospitals in Beijing. Data including sex, age, total amount of TCM prescribed, administration method were obtained (Table 4). Collectively, based on the results from survey and hospital prescriptions, P_95_ values for EF and ED were 90 days per year and 20 years, respectively. Average and P_95_ value for IR were 200 g and 500 g, respectively.

**Table 2:** TCM exposure frequency (days/year)

Administration method	Median	Mean	Maximum	P90	P95
Decoction	8	19.17	183	30	90
Taken orally with water	6	20.12	300	30	150
Taken orally with food	10	57.82	260	180	260
Other	15	104.40	730	455	730
Combination	8	23.36	730	40	90

**Table 3:** TCM daily ingestion ratev (g)

Administration method	Median	Mean	Maximum	P90	P95
Taken orally with water	20	33.66	240	60	130
Raw food	10	43.06	500	45	500
Other ways	10	14.47	30	30	30
Total	80	162.75	4200	300	500

**Table 4:** Statistics on the consumption of TCM prescribed by the hospitals

	Mean	Median	Minimum	Maximum	P75	P90	P95
Total Prescription Dosage	207.2	200.0	4.0	713.0	265.0	332.0	380.8
Maximum dosage of a single medication in a prescription	26.2	30.0	3.0	150.0	30.0	30.0	45.0

### Establishing a database of heavy metals in TCM

From 2016 to 2019, heavy metal contents in commonly used TCM were monitored to establish a database to support risk assessment. Totally 9,300 batches for 140 TCM samples were obtained from wild or cultivated areas, TCM markets, and retail pharmacies. The samples were collected for three years, with each variety having no less than 20 batches per year, to generate a database with wide coverage and strong representativeness. The collection sites encompassed diverse environmental areas in China (82°31′ E to 131°15′ E and 19°07′ N to 48°52′ N). Sample authentication was performed by Dr. Shuai Kang, a scientist experienced in CMM identification. After review by the experts of the steering committee, 14 drug control institutes and scientific research institutes were selected to establish a representative and reliable database of heavy metal contents in TCM, serving for risk assessment. The TCM specimens underwent digestion in a microwave, and heavy metals in TCM were analyzed by ICP-MS [21]. For ensuring the quality of the analysis, blanks, duplicates, spikes, and internal standard recoveries were examined throughout the whole process.

### Drafting the guideline

Two TCM practitioners and one risk assessment expert were tasked to develop a framework for the guideline.

### Contents of the guideline

#### Purpose

The primary purpose of the current guideline is to (i) lay out principles and methods for the risk assessment of heavy metals in TCM, (ii) facilitate the application of scientific approaches in the risk assessment of heavy metals in TCM, (iii) identify high-risk heavy metal indicators based on risk assessment in the final analysis, offering suggestions for risk management.

#### Scope

The guideline provides principles and general procedures for evaluating the risk conferred by heavy metals found in TCM, including lead (Pb), cadmium (Cd), arsenic (As), mercury (Hg) and copper (Cu), etc. The guideline is adequate for both CMM and CPM. In this principle, the risk assessment of TCM as homologous varieties of medicine and food is carried out according to their medicinal effects.

#### Glossary

The glossaries in this guideline include Risk assessment, Deterministic estimate, Lowest-observed-adverse-effect level (LOAEL), No-observed-adverse-effect level (NOAEL), Benchmark dose (BMD), Benchmark dose lower confidence limit (BMDL), Provisional maximum tolerable daily intake (PMTDI), Provisional tolerable monthly intake (PTMI), Provisional tolerable weekly intake (PTWI), Health based guidance value (HBGV), and Lifetime cancer risk (CR), which are listed in Additional file 1: Appendix S1 [22–30].

#### The four steps of risk assessment for heavy metals in TCM

Risk assessment is a strategy focusing on the understanding and evaluation of chemicals’ hazards and exposures, ultimately evaluating the associated health risks. It encompasses four steps, including hazard identification, hazard characterization, exposure assessment and risk characterization.

*Hazard Identification* Hazard Identification aims to identify the types and natures of adverse effects caused by heavy metals in TCM. This step mainly addresses the following two questions: 1) the natures of health hazards that heavy metals in TCM might pose in humans; 2) the conditions in which an identified hazard could be expressed. Using a variety of data, such as animal studies, epidemiological data, and/or structure–activity relationship, the nature of toxicity and involved organs or tissues are determined.

*Hazard Characterization* This second step is a qualitative and, wherever possible, quantitative assessment of the inherent features of heavy metals in TCM potentially causing adverse effects. This includes, if possible, the assessment of a dose–response relationship or the establishment of HBGV (for example, PTWI and PTMI for heavy metals) to provide a quantitative expression of the range of exposure level expected to without appreciable health risk.

*Exposure Assessment* This represents the third step of risk assessment, evaluating the likely intake of heavy metals via TCM for a target population, based on heavy metals’ contents in TCM and clinical consumption data for TCM [31, 32].

For heavy metals in CMM, the equation for deriving the daily exposure level was established as follows:

1 $$\mathrm{Exp}=\frac{\mathrm{EF}\times \mathrm{ED}\times \mathrm{IR}\times \mathrm{C}\times \mathrm{t}}{\mathrm{AT}\times \mathrm{W}}$$

where Exp is the daily exposure level (μg/kg/bw) of CMM; EF represents exposure frequency (day/year) determined from the survey (the provisional P_95_ value of EF is 90 days/year); ED represents exposure duration (20 years for TCM); IR is the daily ingestion rate of CMM (g/day), and its provisional mean and P_95_ values are 200 g and 500 g, respectively, based on the survey; C is heavy metals’ concentration in CMM (mg/kg); t is transfer rate for heavy metals from CMM to decoctions or preparations (%); AT represents the average time of exposure to CMM (365 days/year × 70 years); W represents the body weight (kg) with a mean of 63 kg for adults, and may need to be adjusted for certain patient groups, nationalities,etc.

For heavy metals in CPM, the equation for determining the daily exposure level was established as follows:

2 $$\mathrm{Exp}=\frac{\mathrm{EF}\times \mathrm{Ed}\times \mathrm{IR}\times \mathrm{C}}{\mathrm{AT}\times \mathrm{W}}$$

where Exp is the daily exposure level (μg/kg/bw) of CPM; EF represents exposure frequency (day/year) determined from the survey (provisional P_95_ value of 90 days/year); Ed is exposure duration (years), i.e., 20 years for TCM; IR is daily ingestion rate for CPM (g/day) based on the instructions of the CPM; C is heavy metals’ concentration in CPM (mg/kg); AT is the average time of exposure to CPM (365 days/year × 70 years); W represents the body weight (kg) with a mean of 63 kg in adults, and may need to be adjusted for certain patient groups, nationalities,etc.

*Risk Characterization* This final step of risk assessment integrates Hazard Identification, Hazard Characterization and Exposure Assessment to estimate likely adverse effects in a given population. Risk Characterization estimates the probability of occurrence of known and potential adverse effects exerted by heavy metals. The derived information might be qualitative or quantitative. Different strategies have been applied for the risk characterization of toxic effects viewed as non-carcinogenic or carcinogenic. The Hazard Quotient (HQ) approach is commonly used for non-carcinogenic risk characterization, while the lifetime cancer risk (CR) approach is usually applied for carcinogenic risk characterization.

For non-carcinogenic risk characterization, the HQ approach is commonly used. Here, estimated or measured human exposures are usually compared with the HBGV of heavy metals. The equation for determining HQ was established as follows:

3 $${\text{HQ}} = \frac{{{\text{Exp}} \times 10}}{{\text{ HBGV}}}$$

where Exp is the daily exposure level calculated by Eq. 1 (for CMM) or Eq. 2 (for CPM); 10 is the safety factor, indicating a daily intake of heavy metals from TCM below 10% of their total daily intake; HBGV is usually provided by international regulatory organizations (Additional file 3: Table S1). The HQ approach assumes that in cases with HQ ≤ 1 the level of exposure is unlikely to cause an adverse health effect. In this case, the risk is considered to be acceptable and generally no further risk mitigation measures are needed. On the other hand, if HQ exceeds 1, adverse effects induced by the exposure cannot be excluded, and recommendations are generally put forward for more comprehensive assessments; alternatively, actions are proposed to reduce possible exposures to heavy metals.

For carcinogenic risk characterization, the CR approach is commonly used, which is calculated by the cancer slope factor (CSF) of a given heavy metal carcinogen, e.g., Pb or As. The equation for determining CR has been established as follows [33, 34]:

4 $$\mathrm{CR}=\mathrm{Exp}\times \mathrm{CSF}$$

where Exp is the daily exposure level calculated by Eq. 1 (for CMM) or Eq. 2 (for CPM); CSF represents the oral cancer slope factor, with values for As and Pb of 1.5 and 8.5 × 10^–3^ (mg/kg/day)^−1^, respectively. The CR approach assumes that in case of CR above 10^–4^, the carcinogenic risk of the specific carcinogenic heavy metal over a lifetime is considered to be unacceptable.

#### Complement

In risk assessment, the description of uncertainties should consider the toxicological characteristics of heavy metals in TCM, exposure data, assessment models and hypothetical situations. In addition, technical measures to reduce the uncertainties should also be proposed if necessary. The risk assessment described in the guideline considers TCM serving as therapeutic medicines. When used as dietary supplement or food, assessment parameters for TCM may be altered. In addition, when the medicine is used as a short-term emergency medicine or a long-term chronic medicine, the use frequency and dosage should be adjusted. The risk–benefit balance should be determined based on the clinical purpose.

Health-based guidance levels have been determined by various authoritative organizations, including Joint FAO/WHO Expert Committee on Food Additives (JECFA), USEPA, ATSDR and EFSA. HBGV details are listed in Additional file 3: Table S1.

## Discussion

The results of our database were basically consistent with those reported in the literatures [35, 36]. Based on the database of heavy metals in 9,300 batches of TCM the following conclusion could be obtained: (1) The average levels of Cd, As, Hg, Pb and Cu were 0.29, 0.41, 0.07, 1.35 and 7.47 mg/kg, respectively. (2) The general over-limit ratios of Pb, Cd, As, Hg, and Cu were low; however, contamination in certain TCM cannot be ignored, involving the Pb contents in Morinda officinalis How and the Cd contents in Curcuma phaeocaulis VaL., Lonicera macranthoides Hand. -Mazz., and Lindera aggregata (Sims) Kos-term. Thus, continuous risk monitoring and assessment are recommended. (3) In view of different segments of the herbal plants used in the clinic, leaves and herbs exhibited the highest over-limit ratios, followed by roots and rhizomes. And the over-limit ratios of fruits and seeds were the lowest for all four metals. (4) There were differences in the degree of heavy metal contamination in TCM from different origins. (5) The differences in heavy metal contents between cultivated and wild TCM should not be ignored. (6) Probably due to the fact that animal medicines were at a higher position of the food chain and their main components (including proteins, peptides, and amino acids) chelating with heavy metals, the contents of heavy metals were generally higher than those in herbal medicines.

The risk assessment approaches listed in the guideline are classic deterministic estimates for heavy metals in TCM considering exposure to one heavy metal. In the process of deterministic risk assessment, inputs are represented by single-point estimates. The details of deterministic risk assessment results for heavy metals in TCM using the database established in this study are provided in Additional file 2: Appendix S2. Probabilistic risk assessment considers the effects of uncertainty and variability, and then estimates the range and likelihood of exposure risks, avoiding overestimation or underestimation of health risks and facilitating decision-making [37]. The distribution of parameters instead of a fixed parameter’s value is applied in the probabilistic risk model, enabling a stochastic behavior and probability distribution for the output to be investigated, which should be examined in the future [38]. Additionally, human beings are exposed to multiple pollutants in many ways in real life [39]. Exposure to numerous contaminants through diverse media and pathways is defined as cumulative exposure. Accordingly, cumulative risk assessment is the assessment of health risks produced by cumulative exposure. Cumulative risk assessment strategies for heavy metal contaminants in TCM should be explored in the future to address co-exposure to multiple heavy metals.

Scientific risk assessment is the basis for developing reasonable standard limits. Utilizing the risk assessment approaches in the current guideline, “Guidelines for Establishment of Limit for Harmful Residue of Traditional Chinese Medicine” in the Chinese Pharmacopoeia 2020 edition was established. Compared with the guideline listed in the Chinese Pharmacopoeia 2015 edition, the 2020 edition guideline was especially revised to improve the equation for calculating theoretical limits for exogenous harmful residues, including heavy metals [40]. An advanced equation suitable for TCM was proposed to calculate realistic theoretical limits for heavy metals. Moreover, utilizing the risk assessment approaches in the present guideline and comprehensively considering factors such as public health, economic development, and human cognition, solid safety limit guidance values for all TCM were provided and listed in the 2020 edition of Chinese Pharmacopoeia for the first time.

## Conclusion

In conclusion, this is the first survey-based technical guideline for assessing health risks conferred by heavy metals in TCM in China. The guideline has the ability to scientifically assess the health risks of heavy metals in TCM through a comprehensive application of TCM’s EF, ED and IR from surveys. In addition, safety factors and transfer rates for heavy metals were highlighted. This technical guideline will benefit public health with a standardized risk assessment tool for heavy metals in TCM, advance regulatory standards for heavy metals in TCM and improve human health through scientific TCM usage in the clinic.

## Supplementary Information

**Additional file 1:** Glossary.

**Additional file 2:** Deterministic risk assessment results for heavy metals in TCM.

**Additional file 3: Table S1.** HBGV of heavy metals from different sources.

## Data Availability

Details of data mining, selection, extraction and assessment carried out to support the findings of this study are available from the corresponding author upon request.

## Abbreviations

TCM: Traditional Chinese medicine
CMM: Chinese medicinal materials
CPM: Chinese patent medicine
WHO: World health organization
HBGV: Health based guidance value
JECFA: Joint FAO/WHO Expert Committee on Food Additives
BMD: Benchmark dose
BMDL: Benchmark dose lower confidence limit
LOAEL: Lowest-observed-adverse-effect level
NOAEL: No-observed-adverse-effect level
PMTDI: Provisional maximum tolerable daily intake
PTMI: Provisional tolerable monthly intake
PTWI: Provisional tolerable weekly intake
HQ: Hazard quotient
CR: Lifetime cancer risk
CSF: Cancer slope factor
