# Supplementary material for: Technical guidelines for risk assessment of heavy metals in traditional Chinese medicines
Source: Chin Med. 2023 Jun 7;18:69. doi: 10.1186/s13020-023-00771-3 (PMC10245643; doi:10.1186/s13020-023-00771-3)
Supplement: Supplementary file 1 — Additional file 1: Glossary. [file 13020_2023_771_MOESM1_ESM.docx]

Appendix S1: Glossary

Risk assessment Risk assessment of heavy metals in TCM is a process intended to calculate or estimate the risk to a given target organism, system or (sub)population, including the identification of attendant uncertainties, following exposure to heavy metals in TCM, taking into account the inherent characteristics of heavy metals of concern as well as the characteristics of the specific target system. It is the first component in a risk analysis process.

Deterministic estimate Deterministic estimate of heavy metals in TCM is an estimate that is based on a single value for each model input and a corresponding individual value for a model output, without quantification of the cumulative probability or, in some cases, plausibility of the estimate with respect to the real-world system being modeled. This term is also used to refer to a model for which the output is uniquely specified based on selected single values for each of its inputs.

Lowest-observed-adverse-effect level (LOAEL) LOAEL is the lowest concentration or amount of a substance, found by found by experiment or observation, that causes an adverse alteration of morphology, functional capacity, growth, development or lifespan of the target organism distinguishable from normal (control) organisms of the same species and strain under the same defined conditions of exposure.

No-observed-adverse-effect level (NOAEL) NOAEL is the greatest concentration or amount of a substance, found by experiment or observation, that causes no adverse alteration of morphology, functional capacity, growth, development or lifespan of the target organism distinguishable from those observed in normal (control) organisms of the same species and strain under the same defined conditions of exposure.

Benchmark dose (BMD) BMD is a dose of a substance associated with a specified low incidence of risk, generally in the range of 1-10%, of a health effect.

Benchmark dose lower confidence limit (BMDL) BMDL is the lower boundary of the confidence interval (usually 95%) on the BMD. The BMDL accounts for the uncertainty in the estimate of the dose-response that is due to characteristics of the experimental design, such as sample size. The BMDL can be used as the point of departure (POD) for derivation of a health-based guidance value or a margin of exposure.

Provisional maximum tolerable daily intake (PMTDI) PMTDI is the reference value, established by the Joint FAO/WHO Expert Committee on Food Additives (JECFA), used to indicate the safe level of intake of a contaminant with no cumulative properties. Its value represents permissible human exposure as a result of the natural occurrence of the substance in food and drinking-water. In the case of trace elements that are both essential nutrients and unavoidable constituents of food, a range is expressed, the lower value representing the level of essentiality and the upper value the PMTDI. The tolerable intake is generally referred to as “provisional” as there is often a paucity of data on the consequences of human exposure at low levels, and new data may result in a change to the tolerable level.

Provisional tolerable monthly intake (PTMI) PTMI is an end-point used by JECFA for a food contaminant such as heavy metals, with cumulative properties that has a very long half-life in the human body. Its value represents permissible human monthly exposure to a contaminant unavoidably associated with otherwise wholesome and nutritious foods.

Provisional tolerable weekly intake (PTWI) PTWI is the end-point used by JECFA for food contaminants such as heavy metals, with cumulative properties. Its value represents permissible human weekly exposure to those contaminants unavoidably associated with the consumption of otherwise wholesome and nutritious foods.

Health based guidance value (HBGV) A numerical value derived by dividing a point of departure (NOAEL, BMD or BMDL) by a composite uncertainty factor to determine a level that can be ingested over a defined time period (e.g. lifetime or 24 h) without appreciable health risk. Related terms: PMTDI, PTMI, PTWI.

The lifetime cancer risk (CR) CR is described as the probability of a person developing cancer throughout the lifetime as a result of exposure to specific carcinogenic heavy metal.
