# Supplementary material for: Technical guidelines for risk assessment of heavy metals in traditional Chinese medicines
Source: Chin Med. 2023 Jun 7;18:69. doi: 10.1186/s13020-023-00771-3 (PMC10245643; doi:10.1186/s13020-023-00771-3)
Supplement: Supplementary file 2 — Additional file 2: Deterministic risk assessment results for heavy metals in TCM. [file 13020_2023_771_MOESM2_ESM.pdf]

Pharmacological Research 159 (2020) 104987

Contents lists available at ScienceDirect

Pharmacological Research

journal homepage: www.elsevier.com/locate/yphrs

Innovative health risk assessment of heavy metals in Chinese herbal medicines based on extensive data

Tian-Tian Zuo[a,b,1], Hong-Yu Jin[a,1], Lei Zhang[c,1], Yong-Li Liu[d,1], Jing Nie[e,1], Bi-lian Chen[f,1], Cui-fen Fang[f,1], Jian Xue[g,1], Xue-yan Bi[h,1], Li Zhou[i,1], Ming-rui Shen[j,1], Shang-mei Shi[j,1,**], Shuang-Cheng Ma[b,1,*]

[a] National Institutes for Food and Drug Control, Beijing, 100050, China
[b] Xi'an Jiaotong University, Xi'an, 710049, China
[c] China National Center for Food Safety Risk Assessment, Beijing, 100082, China
[d] Hebei Institute for Drug Control, Shijiazhuang, 050011, China
[e] Hubei Institute for Drug Control, Wuhan, 430075, China
[f] Zhejiang Institute for Food and Drug Control, Hangzhou, 310052, China
[g] Institute of Medicinal Plant Development, Beijing, 100193, China
[h] Heilongjiang Institute for Drug Control, Harbin, 150088, China
[i] National Resource Center for Chinese Materia Medica, China Academy of Chinese Medical Sciences, Beijing, 100700, China
[j] Chinese Pharmacopeia Commission, Beijing, 100061, China

ARTICLE INFO

Keywords:
Heavy metals
Chinese herbal medicines (CHMs)
Guideline
Risk assessment
Safety standard

ABSTRACT

In the present study, the concentrations of lead (Pb), cadmium (Cd), arsenic (As), mercury (Hg), and copper (Cu) in 2245 batches of Chinese herbal medicines (CHMs) were measured using inductively coupled plasma-mass spectroscopy (ICP-MS). We developed a risk assessment strategy that assessed the heavy metal-associated health risk of CHMs based on our large dataset. Using a combination of the mean and 95th percentile (P95) values of the chronic daily intake (CDI), hazard quotient (HQ), hazard index (HI), and lifetime cancer risk (CR), the health risks of the average exposure population and the high exposure population were estimated, respectively. To obtain a precise and realistic risk assessment, the exposure frequency and exposure duration were determined using questionnaire data from 20,917 randomly selected volunteers. Additionally, given the specific ingestion characteristics of CHMs, the safety factor and the transfer rates of heavy metals were highlighted as well. The concentrations of Pb, Cd, As, Hg, and Cu in 2245 batches of CHMs were 1.566, 0.299, 0.391, 0.074, and 8.386 mg/kg, respectively. The mean HI values indicated that consumption of most CHMs would not pose an unacceptable health risk to the average exposure population, except for argy wormwood leaf (1.326), morinda root (2.095), plantain herb (1.540), chrysanthemum flower (1.146), and Indian madder root (2.826). In addition, CR assessment for Pb and As revealed that, for the average exposure population, the risk of developing cancers was lower than the acceptable levels (1×10^{-4}) in the clinic. However, the P95 of the HI and CR values indicated that more attention should be paid to the systemic effects of CHMs in terms of both non-carcinogenic and carcinogenic health risks for the high exposure population. Furthermore, in order to serve population health better, national and international guidelines have now been established. The risk assessment strategy developed in this study is the first of its kind, and contributed to the risk assessment, guidelines, and safety standards for heavy metals in CHMs.

Abbreviations: TCMs, traditional chinese medicines; CHMs, chinese herbal medicines; MeHg, methylmercury; CR, lifetime cancer risk; CSF, cancer slope factor; CDI, chronic daily intake; ICP-MS, inductively coupled plasma mass spectrometry; HQ, hazard quotient; HI, hazard index; WHO, World health organization

* Corresponding author at: National Institutes for Food and Drug Control, No. 2 Tiantan Xili, Dongcheng District, Beijing, 100050, China.
** Corresponding author at: Chinese Pharmacopeia Commission, Fahua Nanli, Gymnasium Road, Dongcheng District, Beijing, 100061, China.
E-mail addresses: zuotiantian2011@163.com (T.-T. Zuo), jhyu@nifdc.org.cn (H.-Y. Jin), zhanglei@cfsa.net.cn (L. Zhang), liuyongli2008@126.com (Y.-L. Liu), niejingwh@sina.com (J. Nie), zsyonly@hotmail.com (B.-l. Chen), fcf0507@126.com (C.-f. Fang), jxue@implad.ac.cn (J. Xue), 65554629@qq.com (X.-y. Bi), zhouli3131@163.com (L. Zhou), shenmingrui@chp.org.cn (M.-r. Shen), ssm@chp.org.cn (S.-m. Shi), masc@nifdc.org.cn (S.-C. Ma).
[1] These authors contributed equally to this work.

https://doi.org/10.1016/j.phrs.2020.104987
Received 13 April 2020; Received in revised form 26 May 2020; Accepted 28 May 2020
Available online 05 June 2020
1043-6618/ © 2020 Elsevier Ltd. All rights reserved.

Table 1
Sample collection information in the present study.

Type	Category	Batch number	Location
Argy Wormwood Leaf	Radix and rhizome	42	Hubei, Chongqing, Zhejiang, Guangxi, Guizhou, Hunan, Fujian, Jiangsu, Shaanxi, Henan, Anhui
Morinda Root	Radix and rhizome	72	Guangdong, Sichuan, Fujian
Peppermint	Leaf	81	Hebei, Yunnan, Jilin, Anhui, Henan, Hubei, Guangxi, Shandong, Jiangsu, Jiangxi, Sichuan
Plantain Herb	Herb	68	Sichuan, Henan, Hubei, Anhui, Shanxi, Guizhou, Guangxi, Jiangxi, Hunan)
Szechwan Lovage Rhizome	Radix and rhizome	61	Sichuan
Rhubarb	Radix and rhizome	50	Gansu, Sichuan, Qinghai
Chinese Date	Fruit	71	Shanxi, Shaanxi, Henan, Hebei, Xinjiang
Chinese Angelica	Radix and rhizome	44	Gansu, Hubei
Tangshen	Radix and rhizome	132	Gansu, Heilongjiang
Zedoray Rhizome	Radix and rhizome	140	Guangxi, Zhejiang, Sichuan, Vietnam, Yunnan
Barbary Wolfberry Fruit	Fruit	62	Xinjiang, Ningxia, Qinghai, Hebei, Beijing
Snowbellleaf Tickclover Herb	Herb	60	Guangdong, Guangxi, Hainan
Safflower	Flower	64	Sichuan, Yunnan, Xinjiang, Inner Mongolia, Henan, Hubei, Beijing
Golden Thread	Radix and rhizome	147	Chongqing, Sichuan, Hubei
Baical Skullcap Root	Radix and rhizome	40	Hebei, Shanxi
Japanese Honeysuckle Flower	Flower	74	Henan, Shandong, Hebei, Hubei, Anhui
Chrysanthemum Flower	Flower	79	Anhui, Hebei, Shandong, Henan, Jiangsu, Sichuan, Zhejiang, Hubei
Bitter Apricot Seed	Seed	62	Gansu, Hebei, Shanxi, Inner Mongolia, Ningxia
Common Coltsfoot Flower	Flower	60	Gansu, Hebei, Shaanxi, Hubei, Hunan, Inner Mongolia
Longtube Ground Ivy Herb	Herb	64	Anhui, Hubei, Henan, Jiangsu, Sichuan, Hunan, Jiangxi
Southern Magnoliavine Fruit	Fruit	63	Henan, Shaanxi, Hubei, Sichuan, Shanxi
Indian Madder Root	Radix and rhizome	84	Sichuan, Shanxi, Hebei, Henan, Yunnan, Heilongjiang, Shaanxi, Shandong
Ginseng	Radix and rhizome	50	Jilin, Heilongjiang
Senchi	Radix and rhizome	65	Yunnan, Guangxi
Perilla Fruit	Fruit	61	Hubei, Sichuan, Jiangsu, Henan, Anhui, Guangxi, Guangdong, Gansu, Hubei, Henan, Shanxi, North Korea
Cape Jasmine Fruit	Fruit	63	Hubei, Henan, Fujian, Jiangxi, Hunan, Sichuan, Zhejiang
Turmeric Root Tuber	Radix and rhizome	69	Sichuan, Guangxi, Zhejiang, Guangdong
Chinese Magnoliavine Fruit	Fruit	61	Jilin, Shaanxi, Liaoning, Hebei, Hubei
Peach Seed	Seed	63	Henan, Gansu, Shaanxi, Hubei, Shanxi, Hebei, Ningxia, Jilin, Inner Mongolia, Shandong
Spine Date Seed	Seed	63	Shandong, Shanxi, Henan, Hebei, Liaoning
Dyers Woad Leaf	Leaf	61	Henan, Anhui, Hebei, Heilongjiang, Guangxi
Perilla Leaf	Leaf	69	Guangxi, Henan, Hubei, Hebei, Sichuan, Jiangsu, Hunan, Guangdong, Anhui, Zhejiang

1. Introduction

Global attention has been directed towards heavy metals because of the negative effects and deterioration they cause in living creatures including humans [1,2]. Heavy metal pollution is a severe problem, owing to its environmental ubiquity, non-biodegradability, persistence, and toxicity, even at trace levels [3–6]. Natural sources involving bedrock erosion and volcanism, as well as anthropogenic activities such as agricultural activities, industrialization, and urbanization have been identified as contributors to heavy metal pollution [7–11]. Heavy metals such as lead (Pb), cadmium (Cd), arsenic (As), mercury (Hg) are considered detrimental to human health, even at trace concentrations [12,13]. Previous studies have reported that these heavy metals exert hazardous effects by affecting cell signaling, protein folding, ionic transportation, and DNA functions [14,15]. Pb is one of the most toxic metals and continuous exposure to Pb affects the normal functioning of the reproductive system and kidneys, as well as the nervous system, causing hyperactivity, anemia, and fatigue [16–20]. Chronic exposure to Cd results in declined cognitive capacity, fractures, reproductive deficiencies, and diabetes [21–24]. As is a ubiquitous metalloid, that is carcinogenic and mutagenic without a threshold. Long-term exposure to As may increase the risk of cancer in the liver, bladder, and lungs, as well as kidney dysfunction, immune system diseases, excessive stillbirth, muscle spasms, and peripheral neuritis [25–28]. Methylmercury (MeHg), the most toxic chemical form of Hg, is highly poisonous to the central nervous system (CNS), triggering lesions in multiple organs throughout an individual's lifetime [29–33]. Cu is an essential trace element for human health; however, non-carcinogenic toxic symptoms may occur in humans after excessive consumption of Cu [34,35].

Chinese herbal medicine (CHM) is an important type of traditional Chinese medicine (TCM) that has a long history of use, and has been described in various traditional medicine documents, including "Shen Nong's Materia Medica", 1800 years ago. CHMs have demonstrated good pharmacological activities, excellent curative effects, and are used extensively in China and around the world. According to statistical data from the World Health Organization (WHO), up to 80 % of the population worldwide has used CHMs [36]. However, CHMs may be polluted by heavy metals during cultivation, harvesting, and processing. The ingestion of CHMs contaminated with heavy metals through the food chain leads to the accumulation of these harmful contaminants in living organisms, including human beings, and poses a serious risk to public health.

Previously, studies on risk assessments on food or environments contaminated by heavy metals have been conducted [37–49]. However, there are distinct differences between CHMs and typical food, and to the best of our knowledge, little data regarding health risk assessment approaches applicable to CHMs are available. Additionally, previous studies often focused on the risks of only limited types of CHMs [50–52]. However, no systematic research has been performed on the health risks of heavy metals using a large dataset that contains various types of CHMs. Thus, their general risk levels have yet to be investigated. Given the globalization and expanded commercialization of CHMs, as well as their associated concerns for human health, it is essential to obtain a general conclusion about heavy metal levels in CHMs and their associated health risks. Considering the distinct differences between CHMs and food, as well as the research gap in health risk strategies that are applicable to CHMs, developing a strategy for CHM risk assessment is essential. Doing so would allow one to scientifically evaluate the health risks of heavy metals and guide the clinical use of CHMs. Therefore, the purposes of this study were as follows: (1) to monitor the levels of Pb, Cd, As, Hg, and Cu in CHMs; (2) to develop an effective approach for CHM risk assessment, in order to scientifically evaluate the associated non-carcinogenic and lifetime cancer risks to human health, which would be done using chronic daily intake (CDI), hazard quotient (HQ), hazard index (HI), and cancer risk (CR) scales. (3) To establish guidelines and provide suggestions for heavy metal

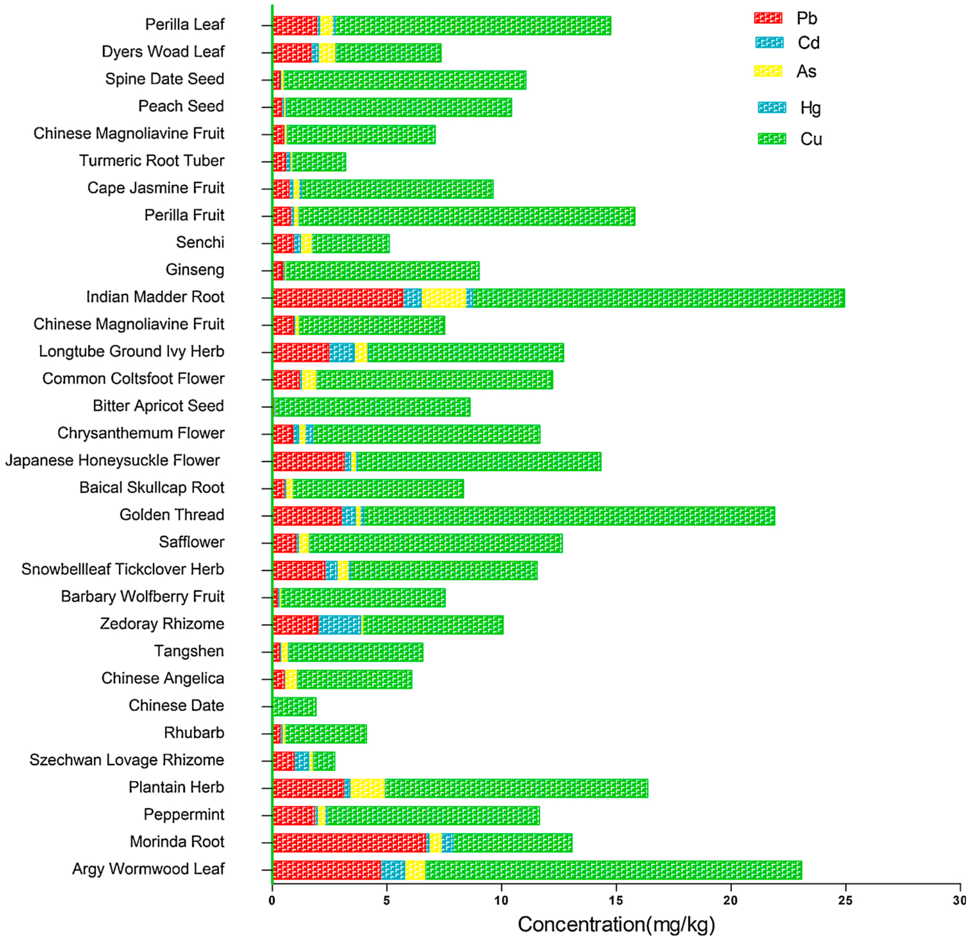

Fig. 1. Mean concentrations of heavy metals in CHMs.

safety standards that are applicable to CHMs, in order to improve the standardization of CHMs and use them safely in clinics.

2. Material and methods

2.1. Sample collection

A relatively complete investigation of CHM categories from major or artificial cultivation areas, TCM markets, and retail pharmacies was conducted to determine the detection samples. Representative and commonly used CHM samples were collected from these areas. Based on the parts of herbal plants used in clinics, CHMs were divided into seven categories, including radix and rhizome, leaf, flower, fruit, seed, herb, and others. At least three types of samples were collected for each available CHM category. A total of 2245 batches of 32 types of CHMs were collected from the city of Beijing and provinces of Xinjiang, Inner Mongolia, Qinghai, Sichuan, Heilongjiang, Gansu, Yunnan, Guangxi, Hunan, Shaanxi, Hebei, Jilin, Hubei, Guangdong, Guizhou, Jiangxi, Henan, Shanxi, Shandong, Liaoning, Anhui, Fujian, Jiangsu, Zhejiang, Chongqing, Ningxia, and Hainan (Table 1). The collecting sites reflected different environmental areas in China (between 86°37′ E and 130°16′ E and 20°07′ N to 46°56′ N). The samples were authenticated by Dr. Kunzi Yu, an associate researcher on the identification of medicinal materials. Voucher specimens were deposited in National Institutes for Food and Drug Control (NIFDC), Beijing, China.

2.2. Sample preparation

The samples were digested using a microwave digestion system (CEM Corporation, Matthews, NC, USA). Approximately 0.5 g of the sample was placed into a microwave digestion tube and then 8.0 mL HNO_3 was added to the sample for digestion. The microwave digestion process was as follows: heating for 3 min to 120 °C and keeping for 3 min, heating for 2 min to 150 °C and keeping for 3 min, heating for 2 min to 180 °C and keeping for 12 min. After removing the excess acid, the digested solution was then diluted to 50.0 mL with deionized water, prior to determination.

2.3. Sample analysis, quality assurance, and quality control

During the determination process, the Agilent 7700X ICP-MS (Agilent 7700X, Agilent Technologies Co., USA) was used to measure the contents of Pb, Cd, As, Hg, and Cu in each sample. All reagents used were excellent pure. For analytical quality assurance, chemical blanks, spikes, and duplicates were analyzed throughout the analytical process. Each sample was run in duplicate and the final values were an average of the two values. The accuracy of the method was controlled by the mean recovery rate of the samples (n = 9). The mean percentage recoveries of heavy metals in morinda root were as follows: 96 ± 0.62 % (Pb), 92 ± 0.57 % (Cd), 84 ± 3.21 % (As), 89 ± 3.82 % (Hg), and 102 ± 7.83 % (Cu). The standard reference material, citrus leaf, was also evaluated during the determination. The concentrations of Pb, Cd, As, Hg, and Cu in the citrus leaf were 9.5 mg/kg, 1.6×10^{-4} mg/kg, 1.0 mg/kg, 0.14 mg/kg, and 6.8 mg/kg, respectively, which was in agreement with the certified elemental content. The internal standard was added to the blanks, samples, and calibration standard solutions to compensate for the matrix effects and signal drift. The mean recovery rates of the internal standard solution during the measurements ranged

Table 2
Mean and the 95th percentile contents of heavy metals in CHMs.

Types		Contents of heavy metals(mg /kg)				
		Pb	Cd	As	Hg	Cu
Argy Wormwood Leaf	Mean	4.713	1.051	0.884	0.027	16.398
	P95	12.184	3.041	1.542	0.069	46.655
Morinda Root	Mean	6.673	0.151	0.535	0.507	5.198
	P95	15.271	0.285	1.192	3.669	6.403
Peppermint	Mean	1.836	0.116	0.329	0.103	9.254
	P95	4.726	0.490	0.811	0.295	14.774
Plantain Herb	Mean	3.110	0.269	1.506	0.036	11.445
	P95	8.567	0.756	4.261	0.163	21.015
Szechwan Lovage Rhizome	Mean	0.939	0.638	0.173	0.030	0.939
	P95	1.805	1.458	0.348	0.049	26.783
Rhubarb	Mean	0.340	0.086	0.112	0.011	3.551
	P95	1.166	0.266	0.504	0.063	5.514
Chinese Date	Mean	0.024	0.002	0.011	0.001	1.868
	P95	0.108	0.006	0.025	0.008	3.967
Chinese Angelica	Mean	0.512	0.024	0.517	0.025	4.993
	P95	0.899	0.059	1.362	0.092	7.459
Tangshen	Mean	0.313	0.046	0.305	0.031	5.860
	P95	0.597	0.144	0.465	0.149	7.725
Zedoray Rhizome	Mean	1.999	1.843	0.093	0.008	6.120
	P95	7.131	4.636	0.212	0.014	9.927
Barbary Wolfberry Fruit	Mean	0.211	0.075	0.070	0.003	7.177
	P95	0.947	0.205	0.133	0.014	11.088
Snowbellleaf Tickclover Herb	Mean	2.291	0.530	0.472	0.103	8.150
	P95	5.286	1.376	1.105	0.798	12.565
Safflower	Mean	1.027	0.094	0.456	0.031	11.039
	P95	2.370	0.202	1.010	0.218	13.621
Golden Thread	Mean	3.004	0.605	0.216	0.156	17.910
	P95	10.360	1.500	0.537	0.252	36.688
Baical Skullcap Root	Mean	0.517	0.058	0.293	0.012	7.456
	P95	1.532	0.311	1.005	0.052	15.560
Japanese Honeysuckle Flower	Mean	3.126	0.284	0.208	0.088	10.612
	P95	21.004	0.543	0.650	0.528	13.999
Chrysanthemum Flower	Mean	0.888	0.257	0.270	0.345	9.903
	P95	2.107	0.610	0.627	1.999	15.456
Bitter Apricot Seed	Mean	0.053	0.006	0.017	0.035	8.501
	P95	0.186	0.016	0.066	0.291	11.627
Common Coltsfoot Flower	Mean	1.173	0.081	0.647	0.018	10.300
	P95	5.084	0.142	1.659	0.195	12.524
Longtube Ground Ivy Herb	Mean	2.465	1.105	0.556	0.052	8.525
	P95	6.620	14.706	2.864	2.265	11.529
Southern Magnoliavine Fruit	Mean	0.916	0.041	0.182	0.010	6.368
	P95	3.764	0.131	0.277	0.069	11.916
Indian Madder Root	Mean	5.682	0.799	1.935	0.313	16.208
	P95	15.114	1.790	4.190	0.160	41.432
Ginseng	Mean	0.443	0.076	0.054	0.010	8.428
	P95	1.623	0.184	0.130	0.032	11.996
Senchi	Mean	0.924	0.294	0.502	0.017	3.358
	P95	5.106	0.763	1.440	0.039	5.591
Perilla Fruit	Mean	0.787	0.126	0.213	0.026	14.655
	P95	4.921	1.352	1.044	0.245	25.251
Cape Jasmine Fruit	Mean	0.722	0.165	0.260	0.111	8.357
	P95	1.386	0.575	0.960	0.685	13.891
Turmeric Root Tuber	Mean	0.580	0.179	0.087	0.004	2.348
	P95	1.369	0.434	0.258	0.011	3.868
Chinese Magnoliavine Fruit	Mean	0.485	0.020	0.113	0.031	6.454
	P95	3.764	0.131	0.277	0.033	11.916
Peach Seed	Mean	0.396	0.081	0.082	0.052	9.822
	P95	1.451	0.952	0.322	0.606	13.164
Spine Date Seed	Mean	0.332	0.027	0.120	0.019	10.551
	P95	2.428	0.121	1.110	0.145	14.842
Dyers Woad Leaf	Mean	1.689	0.314	0.729	0.023	4.607
	P95	3.925	0.960	2.037	0.105	7.689
Perilla Leaf	Mean	1.932	0.119	0.562	0.125	12.009
	P95	6.309	0.427	1.214	1.013	20.176

from 97.8%–103.2%.

2.4. Questionnaires on the CHM consumption

To ensure a realistic assessment of the health risks associated with CHM intake, a questionnaire-based CHM consumption survey was conducted in 9 provinces (Heilongjiang, Liaoning, Zhejiang, Jiangsu, Shandong, Hubei, Guangdong, Gansu, and Yunnan) and 2 cities (Beijing and Chongqing) in China. A total of 20,917 individuals (9420 men and 11,497 women) were randomly selected as volunteers to take part in the survey. Of these volunteers, 72.63 % of the total volunteers were between 18 and 44 years old, 19.51 % were between 45 and 59 years old, and 7.86 % were over 60 years old 7. The average age of the participants was 37.8 years old. There were 11,358 urban residents and 9559 rural residents. Both face-to-face and prescription survey methods were used to identify the significant factors influencing heavy metal exposure in CHMs.

The volunteers were asked to recall their frequency of CHM consumption. Based on the questionnaire data obtained, the P95 of the duration of CHM intake was 90 days per year; therefore, the frequency of CHMs ingestion in this study was assumed to be 90 days. Based on the questionnaire results, the exposure duration was found to be 20 years. The mean and P95 daily CHM consumption amounts were 200 and 500 g/day, respectively.

2.5. Health risk assessment

2.5.1. Non-carcinogenic risks

Some heavy metals are potentially carcinogenic, while some tend to cause non-carcinogenic health deterioration. Therefore, both the non-carcinogenic risks and lifetime cancer risks of specified heavy metals in CHMs were evaluated in the present study. In order to assess the non-carcinogenic health risks, two parameters were calculated, involving CDI (μg/kg bw/day) [53,54] and HQ, using the following equations:

$$CDI = \frac{EF\times\ Ed\times\ IR\times\ C\times t}{AT\times\ W} \quad (1)$$

where CDI is the chronic daily intake of heavy metals in CHMs; EF is the exposure frequency, which was investigated from the questionnaires and was found to be 90 days/year; Ed represents the exposure duration, which was 20 years based on the questionnaire data; IR is the daily intake rate of CHMs (g/day). According to the statistical data of the questionnaires, the mean and P95 values of IR were 200 and 500 g/day, respectively; C is the content of heavy metals in CHMs (mg/kg); t represents the transfer rate of heavy metals from herbal medicinal materials to decoction or preparations (%). According to our previous study, the transfer rates of heavy metals are < 10 % for most CHMs [55]. Herein, the transfer rate used was assumed to be 10 %; AT is the average exposure time to CHMs, which was equal to 365 days/year × 70 years; W is the average body mass (60 kg). In order to calculate CDI, the mean values of IR and C were adopted for the average exposure population, and P95 value of IR and C were used for the high exposure population.

$$HQ = \frac{CDI\times\ SF\times\ 0.001}{RfD} \quad (2)$$

In Eq. (2), HQ represents the hazard quotient and SF is the safety factor. According to the National Science Foundation (NSF)'s judgment, a 10 % allocation of RfD is included, in order to account for the contribution of dietary supplements as a component of daily food intake, and would not lead to a significant increase in human health risks [56]. Therefore, SF was ten in this study. RfD is the oral reference dose recommended for Pb, Cd, inorganic As, methyl mercury (MeHg), and Cu and was determined to be 0.0035, 0.001, 0.0003, 0.0001, and 0.5 mg/kg bw/day, respectively [57,58]. In cases when the HQ value is > 1, the health risks of the exposure population should not be ignored.

Exposure to more than one pollutant may cause additive effects; therefore, the cumulative health risks posed by heavy metals in CHMs were considered, in order to assess the total health risks from multiple pollutants in this study. The cumulative health risks expressed by HI were calculated based on the following equation:

Table 3
The CDI (μg/kg/d) and HQ for average and high exposure population, respectively, due to ingestion of 32 types of CHMs.

Medicines			Pb	Cd	As	Hg	Cu
Argy Wormwood Leaf	Average exposure population	CDI	0.111	0.025	0.021	0.001	0.385
		HQ	0.316	0.247	0.692	0.063	0.008
	High exposure population	CDI	0.715	0.179	0.091	0.004	2.739
		HQ	2.043	1.785	3.017	0.405	0.055
Morinda Root	Average exposure population	CDI	0.157	0.004	0.013	0.012	0.122
		HQ	0.448	0.035	0.419	1.190	0.002
	High exposure population	CDI	0.896	0.017	0.070	0.215	0.376
		HQ	2.561	0.167	2.332	21.537	0.008
Peppermint	Average exposure population	CDI	0.043	0.003	0.008	0.002	0.217
		HQ	0.123	0.027	0.257	0.242	0.004
	High exposure population	CDI	0.277	0.029	0.048	0.017	0.867
		HQ	0.793	0.288	1.587	1.732	0.017
Plantain Herb	Average exposure population	CDI	0.073	0.006	0.035	0.001	0.269
		HQ	0.209	0.063	1.179	0.085	0.005
	High exposure population	CDI	0.503	0.044	0.250	0.010	1.234
		HQ	1.437	0.444	8.337	0.957	0.025
Szechwan Lovage Rhizome	Average exposure population	CDI	0.022	0.015	0.004	0.001	0.022
		HQ	0.063	0.150	0.135	0.070	4.4×10^{-4}
	High exposure population	CDI	0.106	0.086	0.020	0.003	1.572
		HQ	0.303	0.856	0.681	0.288	0.031
Rhubarb	Average exposure population	CDI	0.008	0.002	0.003	2.6×10^{-4}	0.083
		HQ	0.023	0.020	0.088	0.026	0.002
	High exposure population	CDI	0.068	0.016	0.030	0.004	0.324
		HQ	0.196	0.156	0.986	0.370	0.006
Chinese Date	Average exposure population	CDI	0.001	4.7×10^{-5}	2.6×10^{-4}	2.4×10^{-5}	0.044
		HQ	0.002	4.7×10^{-4}	0.009	0.002	0.001
	High exposure population	CDI	0.006	3.5×10^{-4}	0.001	4.7×10^{-4}	0.233
		HQ	0.018	0.004	0.049	0.047	0.005
Chinese Angelica	Average exposure population	CDI	0.012	0.001	0.012	0.001	0.117
		HQ	0.034	0.006	0.405	0.059	0.002
	High exposure population	CDI	0.053	0.003	0.080	0.005	0.438
		HQ	0.151	0.035	2.665	0.540	0.009
Tangshen	Average exposure population	CDI	0.007	0.001	0.007	0.001	0.138
		HQ	0.021	0.011	0.239	0.073	0.003
	High exposure population	CDI	0.035	0.008	0.027	0.009	0.453
		HQ	0.100	0.085	0.910	0.875	0.009
Zedoray Rhizome	Average exposure population	CDI	0.047	0.043	0.002	1.9×10^{-4}	0.144
		HQ	0.134	0.433	0.073	0.019	0.003
	High exposure population	CDI	0.419	0.272	0.012	0.001	0.583
		HQ	1.196	2.721	0.415	0.082	0.012
Barbary Wolfberry Fruit	Average exposure population	CDI	0.005	0.002	0.002	7.0×10^{-5}	0.169
		HQ	0.014	0.018	0.055	0.007	0.003
	High exposure population	CDI	0.056	0.012	0.008	0.001	0.651
		HQ	0.159	0.120	0.260	0.082	0.013
Snowbellleaf Tickclover Herb	Average exposure population	CDI	0.054	0.012	0.011	0.002	0.191
		HQ	0.154	0.124	0.369	0.242	0.004
	High exposure population	CDI	0.310	0.081	0.065	0.047	0.738
		HQ	0.887	0.808	2.162	4.684	0.015
Safflower	Average exposure population	CDI	0.024	0.002	0.011	0.001	0.259
		HQ	0.069	0.022	0.357	0.073	0.005
	High exposure population	CDI	0.139	0.012	0.059	0.013	0.800
		HQ	0.397	0.119	1.976	1.280	0.016
Golden Thread	Average exposure population	CDI	0.071	0.014	0.005	0.004	0.421
		HQ	0.202	0.142	0.169	0.366	0.008
	High exposure population	CDI	0.608	0.088	0.032	0.015	2.154
		HQ	1.738	0.881	1.051	1.479	0.043
Baical Skullcap Root	Average exposure population	CDI	0.012	0.001	0.007	2.9×10^{-4}	0.175
		HQ	0.035	0.014	0.229	0.028	0.004
	High exposure population	CDI	0.090	0.018	0.059	0.003	0.913
		HQ	0.257	0.183	1.966	0.305	0.018
Japanese Honeysuckle Flower	Average exposure population	CDI	0.073	0.007	0.005	0.002	0.249
		HQ	0.210	0.067	0.163	0.207	0.005
	High exposure population	CDI	1.233	0.032	0.038	0.031	0.822
		HQ	3.523	0.319	1.272	3.099	0.016
Chrysanthemum Flower	Average exposure population	CDI	0.021	0.006	0.006	0.008	0.233
		HQ	0.060	0.060	0.211	0.810	0.005
	High exposure population	CDI	0.124	0.036	0.037	0.117	0.907
		HQ	0.353	0.358	1.227	11.734	0.018
Bitter Apricot Seed	Average exposure population	CDI	0.001	0.00014	4.0×10^{-4}	0.001	0.200
		HQ	0.004	0.001	0.013	0.082	0.004
	High exposure population	CDI	0.011	0.001	0.004	0.017	0.683
		HQ	0.031	0.009	0.129	1.708	0.014

(*continued on next page*)

Table 3 (*continued*)

Medicines			Pb	Cd	As	Hg	Cu
Common Coltsfoot Flower	Average exposure population	CDI	0.028	0.002	0.015	4.2×10^{-4}	0.242
		HQ	0.079	0.019	0.506	0.042	0.005
	High exposure population	CDI	0.298	0.008	0.097	0.011	0.735
		HQ	0.853	0.083	3.246	1.145	0.015
Longtube Ground Ivy Herb	Average exposure population	CDI	0.058	0.026	0.013	0.001	0.200
		HQ	0.165	0.259	0.435	0.122	0.004
	High exposure population	CDI	0.389	0.863	0.168	0.133	0.677
		HQ	1.110	8.632	5.604	13.296	0.014
Southern Magnoliavine Fruit	Average exposure population	CDI	0.022	0.001	0.004	2.3×10^{-4}	0.150
		HQ	0.061	0.010	0.142	0.023	0.003
	High exposure population	CDI	0.221	0.008	0.016	0.004	0.699
		HQ	0.631	0.077	0.542	0.405	0.014
Indian Madder Root	Average exposure population	CDI	0.133	0.019	0.045	0.007	0.381
		HQ	0.381	0.188	1.514	0.735	0.008
	High exposure population	CDI	0.887	0.105	0.246	0.009	2.432
		HQ	2.535	1.051	8.198	0.939	0.049
Ginseng	Average exposure population	CDI	0.010	0.002	0.001	2.3×10^{-4}	0.198
		HQ	0.030	0.018	0.042	0.023	0.004
	High exposure population	CDI	0.095	0.011	0.008	0.002	0.704
		HQ	0.272	0.108	0.254	0.188	0.014
Senchi	Average exposure population	CDI	0.022	0.007	0.012	4.0×10^{-4}	0.079
		HQ	0.062	0.069	0.393	0.040	0.002
	High exposure population	CDI	0.095	0.011	0.008	0.002	0.704
		HQ	0.272	0.108	0.254	0.188	0.014
Perilla Fruit	Average exposure population	CDI	0.018	0.003	0.005	0.001	0.344
		HQ	0.053	0.030	0.167	0.061	0.007
	High exposure population	CDI	0.289	0.079	0.061	0.014	1.482
		HQ	0.825	0.794	2.043	1.438	0.030
Cape Jasmine Fruit	Average exposure population	CDI	0.017	0.004	0.006	0.003	0.196
		HQ	0.048	0.039	0.203	0.261	0.004
	High exposure population	CDI	0.081	0.034	0.056	0.040	0.815
		HQ	0.232	0.338	1.878	4.021	0.016
Turmeric Root Tuber	Average exposure population	CDI	0.014	0.004	0.002	9.4×10^{-5}	0.055
		HQ	0.039	0.042	0.068	0.009	0.001
	High exposure population	CDI	0.080	0.025	0.015	0.001	0.227
		HQ	0.230	0.255	0.505	0.065	0.005
Chinese Magnoliavine Fruit	Average exposure population	CDI	0.011	4.7×10^{-4}	0.003	0.001	0.152
		HQ	0.033	0.005	0.088	0.073	0.003
	High exposure population	CDI	0.221	0.008	0.016	0.002	0.699
		HQ	0.631	0.077	0.542	0.194	0.014
Peach Seed	Average exposure population	CDI	0.009	0.002	0.002	0.001	0.231
		HQ	0.027	0.019	0.064	0.122	0.005
	High exposure population	CDI	0.085	0.056	0.019	0.036	0.773
		HQ	0.243	0.559	0.630	3.557	0.015
Spine Date Seed	Average exposure population	CDI	0.008	0.001	0.003	4.5×10^{-4}	0.248
		HQ	0.022	0.006	0.094	0.045	0.005
	High exposure population	CDI	0.143	0.007	0.065	0.009	0.871
		HQ	0.407	0.071	2.172	0.851	0.017
Dyers Woad Leaf	Average exposure population	CDI	0.040	0.007	0.017	0.001	0.108
		HQ	0.113	0.074	0.571	0.054	0.002
	High exposure population	CDI	0.230	0.056	0.120	0.006	0.451
		HQ	0.370	0.025	0.071	0.059	1.184
Perilla Leaf	Average exposure population	CDI	0.045	0.003	0.013	0.003	0.282
		HQ	0.130	0.028	0.440	0.294	0.006
	High exposure population	CDI	0.370	0.025	0.071	0.059	1.184
		HQ	1.058	0.251	2.375	5.946	0.024

$$HI = HQPb + HQCd + HQAs + HQHg + HQCu \quad (3)$$

2.5.2. Carcinogenic risks

For carcinogenic risk assessment, the lifetime cancer risk (CR) for carcinogenic heavy metals was calculated by the cancer slope factor (CSF). The lifetime CR is described as the probability of a person developing cancer throughout the lifetime as a result of exposure to specific heavy metals. The expression for CR is outlined in the following equation [59,60].

$$CR = CDI \times CSF \times 0.001 \quad (4)$$

CSF is the oral cancer slope factor. The CSF for As and Pb was assumed to be 1.5 and 8.5×10^{-3} $(mg/kg/day)^{-1}$, respectively, according to the Integrated Risk Information System database [61]. Other parameters in Eq. (4) are as shown in Eq. (1) and (2). In general, the acceptableCR standard ranges from 10^{-6} to 10^{-4} [62]. Therefore, if the CR value is $>10^{-4}$, the carcinogenic risk over a lifetime is considered unacceptable.

3. Statistical analysis

The questionnaire data were input with an investigator-developed online system via the Epidata method and was confirmed by a quality controller. Statistical analysis was performed using SPSS 19.0 (IBM Corporation, Armonk, NY, USA). Figures were plotted using GraphPad 5.0 software (San Diego, CA, USA).

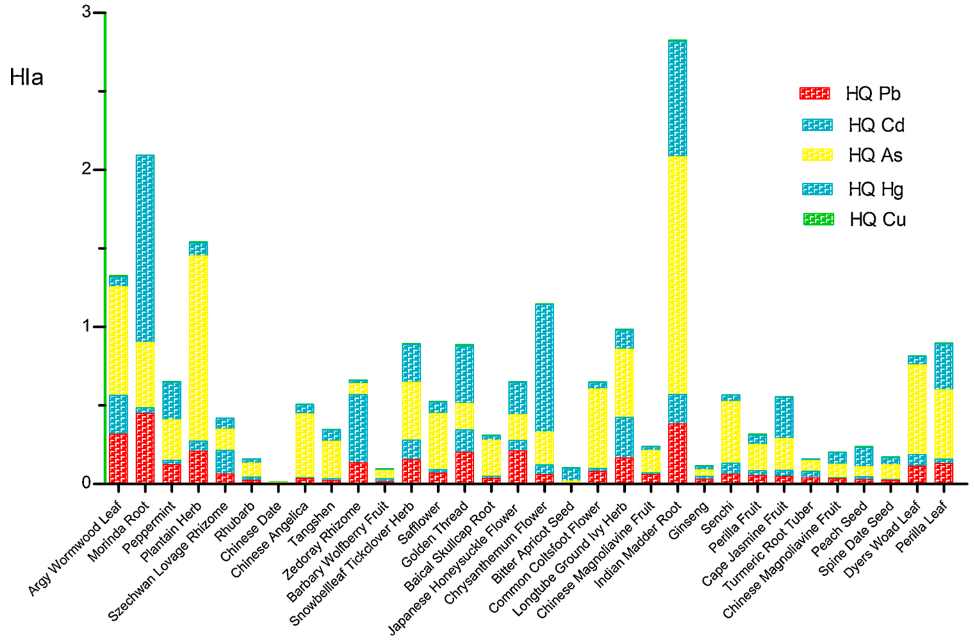

Fig. 2. HI for average exposure population (expressed by HIa).

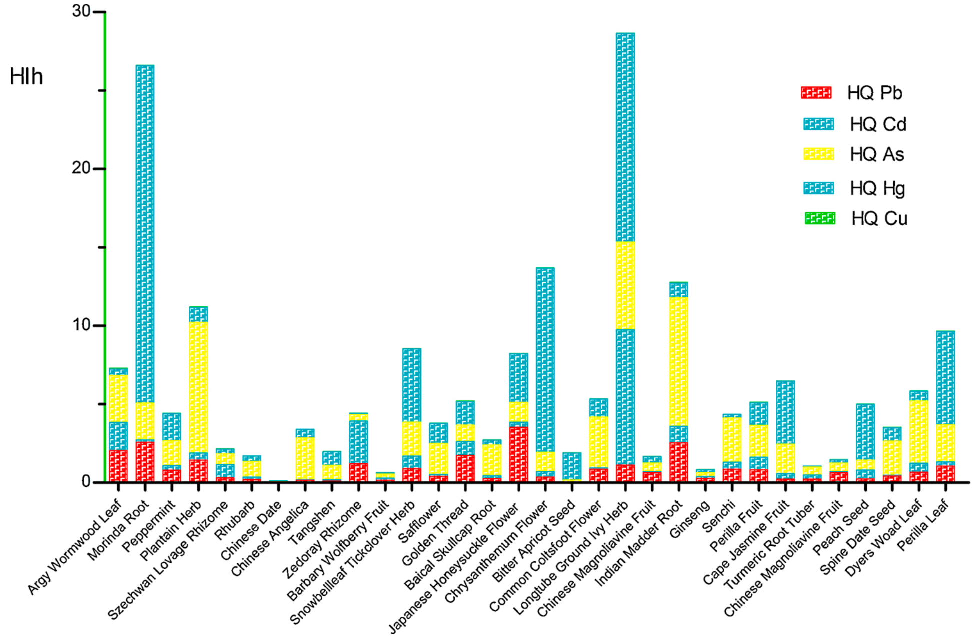

Fig. 3. HI for high exposure population (expressed by HIh).

4. Result and discussion

4.1. Heavy metal contents determined by ICP-MS in CHMs

The maximum permissible limit in the 2020 edition of Chinese Pharmacopoeia for heavy metals in CHMs is 5, 1, 2, 0.2, and 20 mg/kg for Pb, Cd, As, Hg, and Cu, respectively. Levels of heavy metals varied among different species of CHMs, as shown in Fig. 1 and Table 2. Generally, the mean levels of Pb, Cd, As, Hg, and Cu in 2245 batches of CHMs were 1.566, 0.299, 0.391, 0.074, and 8.386 mg/kg, respectively. In Iran, the mean concentrations of Pb, Cd, As, and Cu were determined to be 0.123, 0.034, 0.369, and 3.095 mg/kg, respectively, in rice from a market in Iranshahr [63].The As content was comparable to the average obtained in our research. However, the levels of Pb, Cd, and Cu in the CHMs in the present study were significantly high. In addition, samples from four popular brands of canned fish were analyzed, and the results showed that the contents of Pb, Cd, and Hg (μg/g) were 0.75 ± 0.65, 0.10 ± 0.04, and 0.13 ± 0.05, respectively [64]. The mean concentrations of heavy metals in the f analyzed CHMs varied broadly, from 0.024 to 6.673 mg/kg for Pb, 0.002–1.843 mg/kg for Cd, 0.011–1.935 mg/kg for As, 0.001 to 0.507 mg/kg for Hg, and 0.939–17.910 mg/kg for Cu. The results of the mean values revealed that among the 32 types of CHMs, morinda root, zedoary rhizome, Indian madder root, morinda root, and golden thread were the highest accumulators of Pb, Cd, As, Hg, and Cu, respectively. In a study conducted by Sobhanardakani, the concentrations of Cu in spices (cardamom, curry powder, and turmeric) ranged from 0.05 mg/kg to 1.28 mg/kg, which were significantly lower than the concentrations of CHMs in our current study [65]. In another study, samples from selected foodstuffs in Algeria were analyzed, and the results showed that

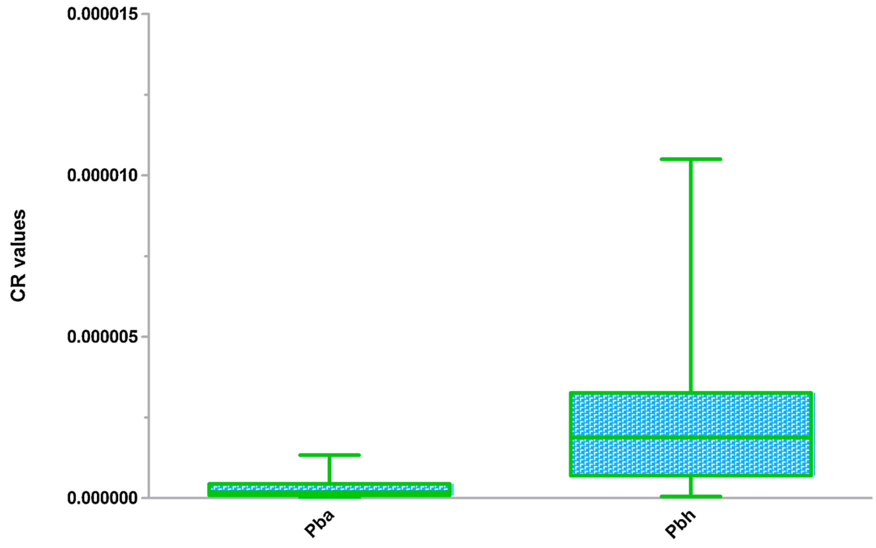

Fig. 4. CR of Pb for average (expressed by Pba) and high (expressed by Pbh) exposure population, respectively.

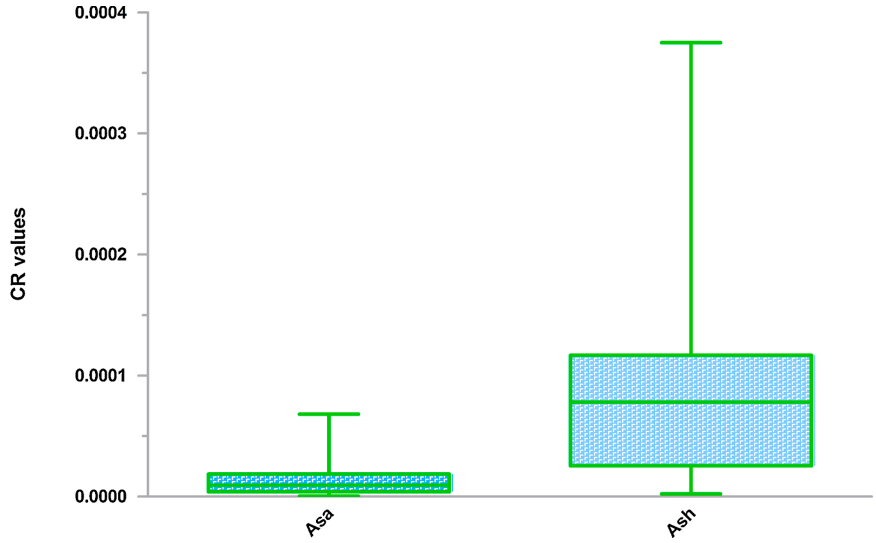

Fig. 5. CR of As for average (expressed by Asa) and high (expressed by Ash) exposure population, respectively.

the contents of Pb and Cu (mg/kg) were within the following ranges: 12.33–39.33 and 4–29.49, respectively [49].

According to the maximum permissible limit for CHMs in the Chinese Pharmacopoeia, qualified rates for Pb, Cd, As, Hg, and Cu in 32 types of analyzed CHMs was 96.24 %, 94.61 %, 97.03 %, 96.28 %, and 96.87 %, respectively (using the formula:

$$qualified\ rate = \frac{number\ of\ samples\ whose\ heavy\ metal\ contents\ were\ lower\ than\ the\ permisible\ \lim it}{total\ number\ of\ samples} \times 100$$

Obviously, Cd was the major risk metal among the five heavy metals, which was consistent with the soil composition in China. From the perspective of different clinical medicinal parts, more attention should be paid to the radix and rhizome category (especially perennial radix and rhizome medicines), as well as the herb category.

4.2. Non-carcinogenic risk assessmentThe CDI values of the average

exposure population revealed that the highest ingestion of Pb, Cd, As, Hg, and Cu occurred with the consumption of morinda root, zedoary rhizome, Indian madder root, morinda root, and golden thread, respectively (Table 3). The highest consumption of Pb, Cd, As, Hg, and Cu in the high exposure population occurred by ingesting Japanese honeysuckle flower, long tube ground ivy herb, plantain herb, morinda root, and argy wormwood leaf, respectively. The CDI of Pb, Cd, As, Hg, and Cu in the average exposure population ranged from 0.001 to 0.157, 4.7×10^{-5} to 0.043, 2.6×10^{-4} to 0.045, 2.4×10^{-5} to 0.012, 0.022 to 0.421 μg/kg/d, respectively. The CDI of Pb, Cd, As, Hg, and Cu in the high exposure population ranged from 0.006 to 1.233, 3.5×10^{-4} to 0.863, 0.001 to 0.250, 4.7×10^{-4} to 0.215, and 0.227–2.739 μg/kg/d, respectively.

Furthermore, to assess the non-carcinogenic health risks associated with chronic exposure to heavy metals in CHMs, HQ values were determined (Table 3). The mean HQ values for the average population's exposure to heavy metals were in the following order : As > Hg > Pb > Cd > Cu, which was consistent with a previous study on the health risks of traditional animal medicines [66]. All HQ values of Pb, Cd, and Cu for a total of 2245 batches of CHMs were lower than 1, which demonstrated that the non-carcinogenic risks caused by Pb, Cd, and Cu in CHMs were within the typical human tolerance range. In another study, the health risks of Pb and Cd associated with fish species

Table 4
CR for As and Pb for average and high exposure population, respectively, due to ingestion of different types of CHMs.

Medicines	Average exposure population		High exposure population	
	Pb	As	Pb	As
Argy Wormwood Leaf	9.41×10^{-7}	3.11×10^{-5}	6.08×10^{-6}	1.36×10^{-4}
Morinda Root	1.33×10^{-6}	1.88×10^{-5}	7.62×10^{-6}	1.05×10^{-4}
Peppermint	3.66×10^{-7}	1.16×10^{-5}	2.36×10^{-6}	7.14×10^{-5}
Plantain Herb	6.21×10^{-7}	5.30×10^{-5}	4.27×10^{-6}	3.75×10^{-4}
Szechwan Lovage Rhizome	1.87×10^{-7}	6.09×10^{-6}	9.01×10^{-7}	3.06×10^{-5}
Rhubarb	6.79×10^{-8}	3.94×10^{-6}	5.82×10^{-7}	4.44×10^{-5}
Chinese Date	4.79×10^{-9}	3.87×10^{-7}	5.39×10^{-8}	2.20×10^{-6}
Chinese Angelica	1.02×10^{-7}	1.82×10^{-5}	4.49×10^{-7}	1.20×10^{-4}
Tangshen	6.25×10^{-8}	1.07×10^{-5}	2.98×10^{-7}	4.09×10^{-5}
Zedoray Rhizome	3.99×10^{-7}	3.28×10^{-6}	3.56×10^{-6}	1.87×10^{-5}
Barbary Wolfberry Fruit	4.21×10^{-8}	2.47×10^{-6}	4.73×10^{-7}	1.17×10^{-5}
Snowbellleaf Tickclover Herb	4.57×10^{-7}	1.66×10^{-5}	2.64×10^{-6}	9.73×10^{-5}
Safflower	2.05×10^{-7}	1.61×10^{-5}	1.18×10^{-6}	8.89×10^{-5}
Golden Thread	6.00×10^{-7}	7.61×10^{-6}	5.17×10^{-6}	4.73×10^{-5}
Baical Skullcap Root	1.03×10^{-7}	1.03×10^{-7}	7.64×10^{-7}	8.85×10^{-5}
Japanese Honeysuckle Flower	6.23×10^{-7}	7.33×10^{-8}	1.05×10^{-7}	5.72×10^{-5}
Chrysanthemum Flower	1.77×10^{-7}	9.51×10^{-9}	1.05×10^{-6}	5.52×10^{-5}
Bitter Apricot Seed	1.06×10^{-7}	5.99×10^{-7}	9.28×10^{-8}	5.81×10^{-6}
Common Coltsfoot Flower	2.34×10^{-7}	2.28×10^{-5}	2.54×10^{-6}	1.46×10^{-4}
Longtube Ground Ivy Herb	4.92×10^{-7}	1.96×10^{-5}	3.30×10^{-6}	2.52×10^{-4}
Southern Magnoliavine Fruit	1.83×10^{-7}	6.41×10^{-6}	1.88×10^{-6}	2.44×10^{-5}
Indian Madder Root	1.13×10^{-6}	6.82×10^{-5}	7.54×10^{-6}	3.69×10^{-4}
Ginseng	8.84×10^{-8}	1.90×10^{-6}	8.10×10^{-7}	1.14×10^{-5}
Senchi	1.84×10^{-7}	1.77×10^{-5}	2.55×10^{-6}	1.27×10^{-4}
Perilla Fruit	1.57×10^{-7}	7.50×10^{-6}	2.46×10^{-6}	9.19×10^{-5}
Cape Jasmine Fruit	1.44×10^{-7}	9.16×10^{-6}	6.92×10^{-7}	8.45×10^{-5}
Turmeric Root Tuber	1.16×10^{-7}	3.06×10^{-6}	6.83×10^{-7}	2.27×10^{-5}
Chinese Magnoliavine Fruit	9.68×10^{-8}	3.98×10^{-6}	1.88×10^{-6}	2.44×10^{-5}
Peach Seed	7.90×10^{-8}	2.89×10^{-6}	7.24×10^{-7}	2.84×10^{-5}
Spine Date Seed	6.63×10^{-8}	4.22×10^{-6}	1.21×10^{-6}	9.77×10^{-5}
Dyers Woad Leaf	3.37×10^{-7}	2.57×10^{-5}	1.96×10^{-6}	1.79×10^{-4}
Perilla Leaf	3.86×10^{-7}	1.98×10^{-5}	3.15×10^{-6}	1.07×10^{-4}

consumed by the Sistan population were investigated, and revealed similar results [67]. The target hazard quotients (THQ) of Pb and Cd were both below 1, revealing an almost safe level of heavy metal contents in the fish [67]. Similarly, the health risk index (HRI) values of Pb, Cd, and Cu suggested that there were no potential health risks for both adults and children from the consumption of *Acipenser persicus* caviar from the Southern Caspian Sea [68]. However, results from this study regarding the health risks associated with As and Hg exposure were notable. As and Hg exist in both inorganic and organic chemical forms in nature; however, different species display different toxicological characteristics. Inorganic arsenic species, including As III and As V have high toxicological effects and have been listed as group I carcinogens without threshold by the International Agency for Research on Cancer (IARC). However, organic arsenic, found mainly as arsenosugar and arsenolipid, is relatively nontoxic. Among the different forms of Hg, MeHg is the most toxic form that is readily accumulated and biomagnified in food chains. With protecting consumers in mind, it was presumed that all the As species in the present study were the most toxic inorganic species and all Hg species were the most hazardous form (MeHg). As a result, 30 types of samples had <1 HQ values for As. However, the HQ values for As in plantain herb (1.179) and Indian madder root (1.514) were larger than 1. A similar scenario was observed in a study conducted in Iran. Djahed et al. investigated the heavy metal contents in rice and found that the calculated HQ for As was larger than one (5.23), indicating non-carcinogenic adverse effects [63]. For Hg, the HQ values in 31 types of CHMs in this study were below 1. However, it was discovered that the HQ value of Hg in morinda root was slightly higher than 1.

The HI values of the average exposure population (HIa) of most of the CHMs were <1, demonstrating that the ingestion of these samples posed no significant risk to human health (Fig. 2). However, the total contribution of Pb, Cd, As, Hg, and Cu led the HIa to be higher than one for argy wormwood leaf (1.326), morinda root (2.095), plantain herb (1.540), chrysanthemum flower (1.146), and Indian madder root (2.826), indicating that people may experience harmful systemic effects related to heavy metals by consuming these five types of CHMs. Generally, As and Hg contributed greatly to the HIa. Argy wormwood leaf, plantain herb, and Indian madder root accumulated a high level of As, contributing primarily to the HIa (>0.5), increasing the HIa to concerning levels. Morinda root and chrysanthemum flower raised the ingestion of Hg and contributed greatly (>0.5) to the HIa. The contributions of different heavy metals to the HIa from argy wormwood leaf can be ranked in the following ascending order: Cu < Hg < Cd < Pb < As. For morinda root, they can be ranked in the following ascending order: Cu < Cd < As < Pb < Hg. For plantain herb: Cu < Cd < Hg < Pb < As. For chrysanthemum flower: Cu < Pb = Cd < As < Hg, and for Indian madder roots: Cu < Cd < Pb < Hg < As.

When it was presumed that all the As and Hg species were the most hazardous chemical forms, the risk assessment results of the high exposure population were not as optimistic as the results of the average exposure population (Fig. 3). Except for Chinese date, barbary wolfberry fruit, and ginseng, the HI values (HIh) of the other 29 types of CHMs were above 1.

4.3. Carcinogenic risk assessment

In the present study, it was assumed that all of the As species in CHMs existed in the most toxic inorganic form. Based on the statistical data (Fig. 4, Fig. 5, and Table 4), it was clear that for the average exposure population, all CR values of Pb and As associated with 32 types of CHMs were lower than 1×10^{-4}. These results suggested that the carcinogenic risks associated with the exposure of Pb and As of all CHM categories analyzed in this study were acceptable. A similar scenario was observed for groundwater in southwestern Nigeria [69], as the CR

values were found to be below acceptable levels. For the high exposure population, all CR values of Pb were $< 1 \times 10^{-4}$; however, the CR values of As associated with argy wormwood leaf, morinda root, plantain herb, Chinese angelica, common coltsfoot flower, long tube ground ivy herb, Indian madder root, sencha, dyers woad leaf, and perilla leaf approached or exceeded 1, and the systemic effects of these CHMs may be of concern. Another study involving an investigation by Fallahzadeh reported that the cancer risk for Pb was high in groundwater samples in Ardakan in 2016 during four seasons [25]. In addition, our previous study on traditional animal medicines revealed that the CR value of As for earthworms was higher than the permissible level [66].

4.4. Guidelines

Various exposure pathways of heavy metals are possible in humans, including inhalation through the nose and mouth, skin contact, and oral intake via the food chain [70]. Compared to direct ingestion, which is the main exposure pathway for humans, other pathways could be negligible. Therefore, to evaluate the potential health risks, the HQ, HI, and CR values of heavy metals in CHMs were determined in the present study. To the best of our knowledge, there is no guideline for assessing the health risks of contaminants in TCMs. Given the distinctive differences between TCMs and food, the National Medical Products Administration (NMPA) systematically designed and took great efforts to determine the crucial parameters for heavy metal risk assessments. This involved determining the exposure frequency and exposure duration from a large amount of questionnaire data, in order to obtain a realistic and refined risk assessment model applicable to TCMs. Notably, the safety factor for TCMs and the transfer rates of heavy metals from herbal medicinal materials to decoctions or preparations were highlighted. For the first time in China, we determined a risk assessment methodology can be used to evaluate the risks from heavy metals in many CHMs. Furthermore, in order to better improve the population's health, a national guideline denoted the "Guideline of risk assessment of exogenous harmful residues in traditional Chinese medicines" has been developed by our study and will be launched in the 2020 edition of the Chinese Pharmacopeia manual [71]. The topics in the guideline mainly include the definition of risk assessments of exogenous harmful residues in TCMs, terms, principles, and basic procedures of the risk assessment. In addition, another guideline named "Technical Guidelines for Risk Assessment of Heavy Metals in Herbal Medicines" has been established and submitted to the International Regulatory Cooperation for Herbal Medicines (IRCH) of the WHO. The contents of the guidelines include the background, scope, glossary, risk assessment procedure, complement, annex, and references for heavy metal risk assessments. These guidelines are significant for practically and scientifically evaluating and controlling possible health risks from heavy metals in TCMs, and provide guidance for the formulation and revision of regulatory standards of heavy metals in TCMs, as well as guide the clinical use of TCMs. B

4.5. Safety standard for heavy metals

Considering the public confidence in the safety of CHMs, the NMPA decided to supervise and take efforts to develop a scientific and practical safety standard for heavy metals in CHMs. A safety standard should be established based on the results from the risk assessment. If the regulatory limit on heavy metals is too strict, it will hinder the economic development of regions that produce CHMs; conversely, if the limit is too relaxed, it may lead to health risks. A practical and scientific heavy metal safety standard for CHMs will enable regions to conserve resources while ensuring public safety, maintaining a balance between health risks and economic progress. Based on the risk assessment model established in this study, the formula for the maximum theoretical limit of heavy metals in the 2020 edition of the Chinese Pharmacopoeia was revised. Furthermore, based on this formula and risk control theory, which considered factors involved in economic development, human cognition, history, and culture, for the first time in China, the heavy metal safety limit guidance values for a diversity of CHMs have been recommended in the Chinese Pharmacopoeia.

4.6. Heavy metal remediation approaches

In the interest of public health, enhanced awareness of ecological environmental protection and remediating areas contaminated with heavy metals is highly recommended in China. The management and control of heavy metal contamination require both international and local efforts. Furthermore, physical, chemical, and biological remediation approaches are encouraged to remove or control heavy metals in contaminated sites [72,73]. Physical remediation includes methods of soil replacement, electro-kinetic remediation, and thermal treatment. Chemical remediation may include chemical stabilization, solidification, and soil washing. Bioremediation is an eco-friendly and cost-effective method compared to the conventional physical and chemical methods. The principles of bioremediation include decreasing the solubility of heavy metals by changing pH, redox reactions, and adsorption from polluted environments [74]. Bioremediation can be realized by microorganisms or plants, by altering biochemical pathways to block heavy metal uptake, converting metals to innocuous forms using enzymes, and reducing intracellular levels of heavy metals using precise efflux systems [74].

4.7. Proposed research on probabilistic assessment

Risk assessment is associated with the uncertainty that may arise from variability in the levels of toxins, uncertainty in the estimation of parameters, a lack of accurate knowledge, and data scarcity [75,76]. Overestimation or underestimation of a risk assessment occurs when these uncertainties are neglected. Generally speaking, there are two approaches to perform a health risk assessment: point estimates and probabilistic approaches. When single-point input parameters are used in a risk assessment, uncertainty may occur. To overcome this problem, the mean and P95 for the CDI, HQ, HI, and CR values were calculated in the present study. Recent studies have reported on the Monte Carlo simulation technique, which applies a probability risk assessment in the field of food and the environment [75–78]. In the analysis of a Monte Carlo simulation, the probabilistic behavior of the risk assessment model is determined, and the uncertainty is described not only by the probability distribution of the inputs, but also by random numbers and statistical methods. Additionally, sensitivity analysis can be accomplished based on Monte Carlo simulation results, in order to analyze the most influential variables determined in the risk assessment [75–78]. For example, Miri et al. assessed the health risks of exposure to polycyclic aromatic hydrocarbons at home and kindergarten for pre-school children using 1-hydroxypyrene as a biomarker. The sensitivity analysis results showed that the concentration of 1-hydroxypyrene had the greatest impact in the risk assessment results [76]. Therefore, a probabilistic approach using Monte Carlo simulations will be explored in our further studies on TCMs.

5. Conclusion

Health risk assessment findings on exposure to heavy metals indicated that consumption of most CHMs would not pose an unacceptable health risk to the average exposure population, except for argy wormwood leaf, morinda root, plantain herb, chrysanthemum flower, and Indian madder root. In addition, a CR assessment for Pb and As revealed that, for the average exposure population, the risk of developing cancer was quite low. However, the P95 of the HI and CR values indicated that more attention should be paid to the systemic effects of CHMs in terms of both non-carcinogenic and carcinogenic health risks for the high exposure population. Given the distinctive

differences between CHMs and food, we built a risk assessment model that is able to scientifically evaluate the risks of heavy metals in diverse and wide-ranging CHMs. This model does this through a comprehensive application of exposure frequency, exposure duration, safety factor, and transfer rates of heavy metals. This model has led to national and international guidelines being established. Furthermore, the formula of maximum limit theoretical value of heavy metals recorded in the 2020 edition of the Chinese Pharmacopoeia has been revised. We hope this study provids insight into the risk assessment and safety standard of heavy metals in CHMs, and sheds light on the standardization and internationalization of TCMs, with the main purpose of improving public health by scientifically using TCMs to treat diverse complex diseases.

6. Authors' contributions

SCM, HYJ and SMS designed the study. TTZ, YLL, JN, BLC, CFF, JX, XYB, LZ conducted the experiments. LZ finished the questionnaire survey. TTZ, HYJ, and MRS analyzed the data. TTZ wrote the manuscript. SCM revised the manuscript. All authors read and approved the final manuscript.

Consent for publication

Not applicable.

Ethics approval and consent to participate

Not applicable.

Availability of data and materials

All data are fully available without restriction.

Funding

This work was financially supported by the 13th 5 Year National Significant New Drugs Creation Feature Subjects (2018ZX09735006) and Project for Medicine and Medical Instruments Review and Approval System Reform (ZG2016-1).

Declaration of Competing Interest

The authors declare that they have no competing interests.

References

[1] R. Kashyap, K.S. Verma, S.K. Uniyal, S.K. Bhardwaj, Geospatial distribution of metal (loid)s and human health risk assessment due to intake of contaminated groundwater around an industrial hub of northern India, Environ. Monit. Assess. 190 (2018) 136.
[2] O. Gündüz, Water quality perspectives in a changing world, Water Qual. Expo. Heal. 7 (2015) 1–3.
[3] Z. Jiang, N. Xu, B. Liu, L. Zhou, J. Wang, C. Wang, B. Dai, W. Xiong, Metal concentrations and risk assessment in water, sediment and economic fish species with various habitat preferences and trophic guilds from Lake caizi, Southeast China, Ecotoxicol. Environ. Saf. 157 (2018) 1–8.
[4] N. Bortey-Sam, S.M. Nakayama, Y. Ikenaka, O. Akoto, E. Baidoo, H. Mizukawa, M. Ishizuka, Health risk assessment of heavy metals and metalloid in drinking water from communities near gold mines in Tarkwa, Ghana, Environ. Monit. Assess. 187 (2015) 1–12.
[5] P.G.C. Emenike, T.I. Tenebe, M. Omeje, D.S. Osinubi, Health risk assessment of heavy metal variability in sachet water sold in Ado-Odo ota, South-Western Nigeria, Environ. Monit. Assess. 189 (2017).
[6] N. Maghakyan, G. Tepanosyan, O. Belyaeva, L. Sahakyan, A. Saghatelyan, Assessment of pollution levels and human health risk of heavy metals in dust deposited on Yerevan's tree leaves (Armenia), Acta. Geochim. 36 (2017) 16–26.
[7] J. Nawab, S. Khan, W. Xiaoping, Chemosphere ecological and health risk assessment of potentially toxic elements in the major rivers of Pakistan: General population vs, Fishermen, Chemosphere. 202 (2018) 154–164.
[8] S. Giri, A.K. Singh, Spatial distribution of metal(loid)s in groundwater of a mining dominated area: recognising metal(loid) sources and assessing carcinogenic and non-carcinogenic human health risk, Int. J. Environ. Anal. Chem. 96 (2016) 1313–1330.
[9] I.T. Tenebe, A. Ogbiye, D.O. Omole, P.C. Emenike, Estimation of longitudinal dispersion co-efficient: a review, Cogent. Eng. 3 (2016).
[10] S. Khan, Q. Cao, Y.M. Zheng, Y.Z. Huang, Y.G. Zhu, Health risks of heavy metals in contaminated soil and food crops irrigated with wastewater in Beijing, China. Environ. Pollut 152 (2008) 686–692.
[11] W.T. Si, J.M. Liu, L. Cai, H. Jiang, C. Zheng, H. He, J. Wang, X. Zhang, Health risks of heavy metals in contaminated farmland soils and spring wheat irrigated with YR water, Bull. Environ. Contam. Toxicol. 94 (2015) 214–219.
[12] H.-J. Hapke, Heavy metal transfer in the food chain to humans, Fertil, Environ. 66 (1996) 431–436.
[13] P.C. Emenike, D.O. Omole, B.U. Ngene, I.T. Tenebe, Potentiality of agricultural adsorbent for the sequestering of metal ions from wastewater, Glob. J. Environ. Sci. Manag. 2 (2016) 411–442.
[14] M. Jaishankar, T. Tseten, N. Anbalagan, B.B. Mathew, K.N. Beeregowda, Toxicity, mechanism and health effects of some heavy metals, Interdiscip. Toxicol. 7 (2014) 60–72.
[15] V. Sirot, T. Guérin, J.L. Volatier, J.C. Leblanc, Dietary exposure and biomarkers of arsenic in consumers of fish and shellfish from France, Sci. Total. Environ. 407 (6) (2009) 1875–1885.
[16] M. Bassil, F. Daou, H. Hassan, O. Yamani, J.A. Kharma, Z. Attieh, J. Elaridi, Lead, cadmium and arsenic in human milk and their socio-demographic and lifestyle determinants in Lebanon, Chemosphere 191 (2018) 911–921.
[17] M.A. Barakat, New trends in removing heavy metals from industrial wastewater, Arab. J. Chem. 4 (2011) 361–377.
[18] D.C. Bellinger, Very low lead exposures and children's neurodevelopment, Curr. Opin. Pediatr. 20 (2008) 172–177.
[19] L. Järup, Hazards of heavy metal contamination, Br. Med. Bull. 68 (1) (2003) 167–182.
[20] X. Chen, K. Wang, Z. Wang, C. Gan, P. He, Y. Liang, T. Jin, G. Zhu, Effects of lead and cadmium co-exposure on bone mineral density in a Chinese population, Bone. 63 (6) (2014) 76–80.
[21] M. Kippler, F. Tofail, J.D. Hamadani, R.M. Gardner, S.M. Grantham-McGregor, M. Bottai, M. Vahter, Early-life cadmium exposure and child development in 5-year-Old girls and boys: a cohort study in rural Bangladesh, Environ. Health. Perspect. 120 (2012) 1462–1468.
[22] A.P. Sanders, B. Claus Henn, R.O. Wright, Perinatal and childhood exposure to cadmium, manganese, and metal mixtures and effects on cognition and behavior: a review of recent literature, Curr. Environ. Heal. Reports. 2 (2015) 284–294.
[23] W.L. Zhang, Y. Du, M.M. Zhai, Q. Shang, Cadmium exposure and its health effects: a 19-year follow-up study of a polluted area in China, Sci. Total. Environ. 470 (2014) 224–228.
[24] Z. Krejpcio, S. Sionkowski, J. Bartela, Safety of fresh fruits and juices available on the Polish market as determined by heavy metal residues, Pol. Environ. Stud. 14 (6) (2005) 877–881.
[25] R.A. Fallahzadeh, M.T. Ghaneian, M. Miri, M.M. Dashti, Spatial analysis and health risk assessment of heavy metals concentration in drinking water resources, Environ. Sci. Pollut. Res. 24 (2017) 24790–24802.
[26] S. Kapaj, H. Peterson, K. Liber, P. Bhattacharya, Human health effects from chronic arsenic poisoning-a review, J. Environ. Sci. Health. A. Tox. Hazard. Subst. Environ. Eng. 41 (2006) 2399–2428.
[27] J. Zukowska, M. Biziuk, Methodological evaluation of method for dietary heavy metal intake, J. Food Sci. 73 (2010) 21–29.
[28] M.F. Hughes, Arsenic toxicity and potential mechanisms of action, Toxicol. Lett. 133 (2002) 1–16.
[29] M. Harada, Minamata disease: methylmercury poisoning in Japan caused by environmental pollution, Crit. Rev. Toxicol. 25 (1995) 1–24.
[30] K. Eto, H. Tokunaga, K. Nagashima, T. Takeuchi, An autopsy case of minamata disease (methylmercury poisoning)–pathological viewpoints of peripheral nerves, Toxicol. Pathol. 30 (2002) 714–722.
[31] S.L. Lee, X. Li, W. Shi, C.N. Cheung, I. Thornton, Metal contamination in urban, suburban, and country park soils of Hong Kong: a study based on GIS and multivariate statistics, Sci. Total. Environ. 356 (2006) 45–61.
[32] K.A. Bjőrnberg, M. Vahter, K. Petersson-Grawé, et al., Methyl mercury and inorganic mercury in Swedish pregnant women and in cord blood: influence of fish consumption, Environ. Health. Perspect. 111 (2003) 637–641.
[33] S.E. Schober, T.H. Sinks, R.L. Jones, P.M. Bolger, M. McDowell, J. Osterloh, E.S. Garrett, R.A. Canady, C.F. Dillon, Y. Sun, C.B. Joseph, K.R. Mahaffey, Blood mercury levels in US children and women of childbearing age, 1999-2000, JAMA. 289 (2003) 1667–1674.
[34] X.D. Zang, H.S.Y. Huang, Z.L. Zhuang, R.S. Chen, Z.Y. Xie, C. Xu, X.M. Mo, The association between serum copper concentrations and cardiovascular disease risk factors in children and adolescents in NHANES, Environ. Sci. Pollut. Res. Int. 25 (2018) 16951–16958.
[35] J. Zhou, J. Liang, Y.M. Hu, W.T. Zhang, H.L. Liu, L.Y. You, W.H. Zhang, M. Gao, J. Zhou, Exposure risk of local residents to copper near the largest flash copper smelter in China, Sci. Total. Environ. 630 (2018) 453–461.
[36] WHO, WHO Traditional Medicine Strategy 2014-2023, Geneva, 2013.
[37] D.O. Omole, I.T. Tenebe, C.P. Emenike, A.S. Umoh, A.A. Badejo, Causes, impact and management of electronic wastes: case study of some nigerian communities, ARPN, J. Eng. Appl. Sci. 10 (2015) 7876.
[38] S. Chinedu, O. Nwinyi, Y. Adetayo, N. Vivienne, Assessment of water quality in canaanland, ota, Southwest Nigeria, Agric. Biol. J. North Am. 2 (2011) 577–583.
[39] J. Chen, H. Wu, H. Qian, Y. Gao, Assessing nitrate and fluoride contaminants in drinking Water and their health risk of rural residents living in a semiarid region of

Northwest China, Expo. Heal. 9 (2017) 183–195.
[40] Z. Zhang, L. Juying, Z. Mamat, Sources identification and pollution evaluation of heavy metals in the surface sediments of bortala River, Northwest China, Ecotoxicol. Environ. Saf. 126 (2016) 94–101.
[41] P.C. Emenike, I.T. Tenebe, P. Jarvis, Fluoride contamination in groundwater sources in southwestern Nigeria: assessment using multivariate statistical approach and human health risk, Ecotoxicol. Environ. Saf. 156 (2018) 391–402.
[42] S. Khan, M. Shahnaz, N. Jehan, S. Rehman, M.T. Shah, I. Din, Drinking water quality and human health risk in Charsadda district, Pakistan. J. Clean. Prod. 60 (2013) 93–101.
[43] A. Alahabadi, M.H. Ehrampoush, M. Miri, H. Ebrahimi Aval, S. Yousefzadeh, H.R. Ghaffari, et al., A comparative study on capability of different tree species in accumulating heavy metals from soil and ambient air, Chemosphere. 172 (2017) 459–467.
[44] S. Nemati, M. Mosaferi, A. Ostadrahimi, A. Mohammadi, Arsenic intake through consumed rice in Iran: markets role or government responsibility, Environ. Health. Perspect. 4 (2014) 180.
[45] Y.Y. Li, H.B. Wang, H.J. Wang, F. Yin, X.Y. Yang, Y.J. Hu, Heavy metal pollution in vegetables grown in the vicinity of a multimetal mining area in Gejiu, China: total concentrations, speciation analysis, and health risk, Environ. Sci. Pollut. Res. 21 (21) (2014) 12569–12582.
[46] O.R. Ghaleno, M. Sayadi, M. Rezaei, Potential ecological risk assessment of heavy metals in sediments of water reservoir case study: chah nimeh of sistan, Proc. Int. Acad. Ecol. Environ. Sci. 5 (2015) 89–96.
[47] M. Yeganeh, M. Afyuni, A.H. Khoshgoftarmanesh, A.R. Soffianian, R. Schulin, Health risks of metals in soil, Water, and Major food crops in hamedan Province, Iran, Hum. Ecol. Risk Assess. 18 (2012) 547–568.
[48] P. Glorennec, J.P. Lucas, A.C. Mercat, A.C. Roudot, B.B.J.E.I. Le, Environmental and dietary exposure of young children to inorganic trace elements, Environ. Int. 97 (2016) 28–36.
[49] A. Cherf, S. Abdoun, O. Gaci, Food survey: levels and potential health risks of chromium, lead, zinc and copper content in fruits and vegetables consumed in Algeria, Food. Chem. Toxicol. 70 (2014) 48–53.
[50] Y. Geng, L. Jiang, H. Jiang, L. Wang, Y. Peng, C. Wang, X. Shi, J. Gu, Y. Wang, J. Zhu, L. Dai, Y. Xu, X. Liu, Assessment of heavy metals, fungicide quintozene and its hazardous impurity residues in medical Panax notoginseng (burk) F.H.Chen root, Biomed Chromatogr. 33 (2019) 4378.
[51] S.Y. Gu, J.Y. Luo, H. Liu, J.J. Wu, W. Qi, Z.W. Fan, M.H. Yang, Determination of arsenic speciation in 17 commonly used traditional Chinese herbal medicines by HPLC-ICP-MS, Chin. J. Chin. Mater. Med. 44 (2019) 3078–3086.
[52] L. Zhou, W. Sheng, Q.X. Hao, L.P. Kang, C.Z. Kang, J. Yang, W.Z. Yang, J.Y. Jiang, L.Q. Huang, L.P. Guo, Bioaccessibility and risk assessment of heavy metals, and analysis of arsenic speciation in *cordyceps sinensis*, Chin. Med. 13 (2018) 40.
[53] C. Roba, C. Roşu, I. Piştea, A. Ozunu, C. Baciu, Heavy metal content in vegetables and fruits cultivated in Baia Mare mining area (Romania) and health risk assessment, Environ. Sci. Pollut. Res. 23 (2016) 6062–6073.
[54] A. Singh, R.K. Sharma, M. Agrawal, F.M. Marshall, Health risk assessment of heavy metals via dietary intake of foodstuffs from the wastewater irrigated site of a dry tropical area of India, Food. Chem. Toxicol. 48 (2010) 611–619.
[55] T.T. Zuo, H.Y. Jin, L.Z, S.C. Ma, The research of transfer rates of heavy metals and harmful elements in tradition Chinese medicines before and after extraction and tiered risk assessment, Chin. J. Pharm. Anal. 37 (8) (2017) 1399–1406.
[56] NSF International, Dietary supplement-standard 173：Metal contaminant accepted level, Ann Arbor (2003) 3–4.
[57] S. Khan, Q. Cao, Y.M. Zheng, Y.Z. Huang, Y.G. Zhu, Health risks of heavy metals in contaminated soil and food crops irrigated with wastewater in Beijing, China, Environ. Pollut. 152 (2008) 686–692.
[58] A. Mahmood, R.N. Malik, Human health risk assessment of heavy metals via consumption of contaminated vegetables collected from different irrigation sources in Lahore, Pakistan, Arab. J. Chem. 7 (2013) 91–99.
[59] S.C. Sofuoglu, P. Kavcar, An exposure and risk assessment for fluoride and trace metals in black tea, J. Hazard. Mater. 158 (2008) 392–400.
[60] H.B. Cao, L. Qiao, H. Zhang, Exposure and risk assessment for aluminum and heavy metals in puerh tea, Sci. Total. Environ. 408 (2010) 2777–2784.
[61] IRIS, Integrated Risk Information System, Available at (2012) https://www.epa.gov/research/integrated-risk-informationsystem-iris-current-assessments-and-recentdevelopments-0.
[62] US Environmental Protection Agency, Guidelines for Carcinogen Risk Assessment, US, (2005).
[63] Djahed, B. Taghavi, M. Farzadkia, S. Norzaee, M. Miri, Stochastic exposure and health risk assessment of rice contamination to the heavy metals in the market of Iranshahr, Iran, Food. Chem. Toxicol. 115 (2018) 405–512.
[64] S. Sobhanardakani, Tuna fish and common kilka: health risk assessment of metal pollution through consumption of canned fish in Iran, J. Consum. Prot. Food. Safety. 12 (2) (2017) 157–163.
[65] S. Sobhanardakani, Potential health risk assessment of Cr, Cu, Fe and Zn for human population via consumption of commercial spices; A case study of hamedan City, Iran, Int. Arch. Health. Sci. 3 (3) (2016) 119–124.
[66] T.T. Zuo, Y.L. Li, H.Z. He, H.Y. Jin, L. Zhang, L. Sun, F. Gao, Q. Wang, Y.J. Shen, S.C. Ma, L.C. He, Refined assessment of heavy metal-associated health risk due to the consumption of traditional animal medicines in humans, Environ. Monit. Assess. 191 (2019) 171.
[67] M. Miri, E. Akbari, A. Amrane, S.J. Jafari, H. Eslami, E. Hoseinzadeh, M. Zarrabi, J. Salimi, M. Sayyad-Arbabi, M. Taghavi, Health risk assessment of heavy metal intake due to Fish consumption in the sistan region, Iran, Environ. Monit. Assess. 189 (11) (2017) 583.
[68] S. Sobhanardakani, L. Tayebi, S. V.Hosseini, Health risk assessment of arsenic and heavy metals (Cd, Cu, Co, Pb, and Sn) through consumption of caviar of acipenser persicus from Southern caspian Sea, Environ. Sci. Pollut. Res. 25 (3) (2018) 2664–2671.
[69] P.C. Emenike, I. Tenebe, N. Ogarekpe, D. Omole, C. Nnaji, Probabilistic risk assessment and spatial distribution of potentially toxic elements in groundwater sources in southwestern Nigeria, Sci. Rep. 9 (1) (2019) 15920.
[70] A. Gholizadeh, M. Taghavi, A. Moslem, A.A. Neshat, M.L. Najafi, A. Alahabadi, E. Ahmadi, H.E. Aval, A.A. Asour, H. Rezaei, S. Gholami, M. Miri, Ecological and health risk assessment of exposure to atmospheric heavy metals, Ecotoxicol. Environ Saf. 184 (109622) (2019).
[71] T.T. Zuo, Y. Wang, L. Zhang, S.M. Shi, M.R. Shen, L.N. Liu, L. Sun, H.Y. Jin, S.C. Ma, Guideline of risk assessment of exogenous harmful residues in traditional Chinese medicines, Chin.J. Pharm. Anal. (39) (2019) 1902–1907.
[72] G.N. Koptsik, Modern approaches to remediation of heavy metal polluted soils: a review, Eurasian. Soil. Sci. 47 (2014) 707–722.
[73] E.O. Dada, K.I. Njoku, A.A. Osuntoki, M.O. Akinola, A review of current techniques of physico-chemical and biological remediation of heavy metals polluted soil, Ethiop J environ. Stud. Manag. 8 (2011) 606–615.
[74] O.B. Ojuederie, O.O. Babalola, Microbial and plant-assisted bioremediation of heavy metal polluted environments: a review, Int. J. Environ. Res. Public. Health. 14 (2017) 1504.
[75] R.A. Fallahzadeh, R. Khosravi, B. Dehdashti, E. Ghahramani, F. Omidi, A. Adli, M. Miri, Spatial distribution variation and probabilistic risk assessment of exposure to chromium in Ground Water supplies; A case study in the East of Iran, Food. Chem. Toxicol. 115 (2018) 260–266.
[76] M. Miri, A. Alahabadi, M.H. Ehrampoush, H.R. Ghaffari, M.J.Z. Sakhvidi, M. Eskandari, A. Rad, M.H. Lotfi, M.H. Sheikhha, Environmental determinants of polycyclic aromatic hydrocarbons exposure at home, at kindergartens and during a commute, Environ. Int. 118 (2018) 266–273.
[77] R.A. Fallahzadeh, M.T. Ghaneian, M. Miri, M. M.Dashti, Spatial analysis and health risk assessment of heavy metals concentration in drinking water resources, Environ. Sci. Pollut. Res. 24 (32) (2017) 24790–24802.
[78] A. Gholizadeh, M. Mokhtari, N. Naimi, B. Shiravand, M.H. Ehrampoush, M. Miri, A. Ebrahimi, Assessment of corrosion and scaling potential in groundwater resources; A case study of Yazd-Ardakan Plain, Iran, Groundwater for Sustainable Development 5 (2017) 59–65.
