# Supplementary material for: Technical guidelines for risk assessment of heavy metals in traditional Chinese medicines
Source: Chin Med. 2023 Jun 7;18:69. doi: 10.1186/s13020-023-00771-3 (PMC10245643; doi:10.1186/s13020-023-00771-3)
Supplement: Supplementary file 3 — Additional file 3: Table S1. HBGV of heavy metals from different sources. [file 13020_2023_771_MOESM3_ESM.docx]

**Table S1.** HBGV of heavy metals from different sources

| Source | Arsenic (As) | Cadmium (Cd) | Lead (Pb) | Mercury (Hg) |
| --- | --- | --- | --- | --- |
| JECFA | 15 μg/kg bw/week (Withdraw, 2011)；  BMDL_0.5_: 3 µg/kg bw/day (lung cancer) | 25 μg/kg bw/month | 25 μg/kg bw/week (Withdraw, 2011)  POD: 1.3 µg/kg bw/d for 1 mmHg increase in blood pressure | Total Hg:4μg/kg bw/week;  MeHg:1.6μg/kg bw/week |
| US EPA | 0.3 μg/kg bw/day | 1μg/kg bw/day | 3.5μg/kg bw/day | MeHg:  0.1μg/kg bw/day |
| ESFA | -- | 2.5 μg/kg bw/week | -- | Total Hg:4.2 μg/kg bw/week |
| ATSDR | 0.3 μg/kg bw/day | 0.1 μg/kg bw/day | -- | MeHg:  0.3 μg/kg bw/day |
